# YAP1 and WWTR1 expression inversely correlates with neuroendocrine markers in Merkel cell carcinoma

**DOI:** 10.1172/JCI157171

**Published:** 2023-03-01

**Authors:** Thomas C. Frost, Ashley K. Gartin, Mofei Liu, Jingwei Cheng, Harita Dharaneeswaran, Derin B. Keskin, Catherine J. Wu, Anita Giobbie-Hurder, Manisha Thakuria, James A. DeCaprio

**Affiliations:** 1 Program in Virology, Graduate School of Arts and Sciences, Harvard University, Cambridge, Massachusetts, USA.; 2 Department of Medical Oncology and; 3 Department of Data Sciences, Dana-Farber Cancer Institute (DFCI), Boston, Massachusetts, USA.; 4 Department of Molecular, Cellular, and Biomedical Sciences, College of Life Sciences and Agriculture, University of New Hampshire, Durham, New Hampshire, USA.; 5 Merkel Cell Carcinoma Center of Excellence, Dana-Farber/Brigham Cancer Center, Boston, Massachusetts, USA.; 6 Department of Medicine, Brigham and Women’s Hospital, Harvard Medical School, Boston, Massachusetts, USA.; 7 Broad Institute of MIT and Harvard, Cambridge, Massachusetts, USA.; 8 Translational Immunogenomics Laboratory, DFCI, Boston, Massachusetts, USA.; 9 Department of Computer Science, Metropolitan College, Boston University, Boston, Massachusetts, USA.; 10 Section for Bioinformatics, Department of Health Technology, Technical University of Denmark, Lyngby, Denmark.; 11 Department of Dermatology, Brigham and Women’s Hospital, Harvard Medical School, Boston, Massachusetts, USA.

**Keywords:** Dermatology, Oncogenes, Transcription, Tumor suppressors

## Abstract

**Background:**

Merkel cell carcinoma (MCC) is an aggressive neuroendocrine (NE) skin cancer caused by severe UV-induced mutations or expression of Merkel cell polyomavirus (MCPyV) large and small T antigens (LT and ST). Despite deep genetic differences between MCPyV-positive and -negative subtypes, current clinical diagnostic markers are indistinguishable, and the expression profile of MCC tumors is, to our knowledge, unexplored.

**Methods:**

Here, we leveraged bulk and single-cell RNA-Seq of patient-derived tumor biopsies and cell lines to explore the underlying transcriptional environment of MCC.

**Results:**

Strikingly, MCC samples could be separated into transcriptional subtypes that were independent of MCPyV status. Instead, we observed an inverse correlation between a NE gene signature and the Hippo pathway transcription factors Yes1-associated transcriptional regulator (YAP1) and WW domain–containing transcriptional regulator 1 (WWTR1). This inverse correlation was broadly present at the transcript and protein levels in the tumor biopsies as well as in established and patient-derived cell lines. Mechanistically, expression of YAP1 or WWTR1 in a MCPyV-positive MCC cell line induced cell-cycle arrest at least in part through TEA domain–dependent (TEAD-dependent) transcriptional repression of MCPyV LT.

**Conclusion:**

These findings identify what we believe to be a previously unrecognized heterogeneity in NE gene expression within MCC and support a model of *YAP1/WWTR1* silencing as essential for the development of MCPyV-positive MCC.

**Funding:**

US Public Health Service grants R35CA232128, P01CA203655, and P30CA06516.

## Introduction

### MCC can be defined by viral status and NE marker expression.

Merkel cell carcinoma (MCC) is a neuroendocrine (NE) carcinoma of the skin with 2 etiological subtypes distinguished by the presence or absence of the integrated Merkel cell polyomavirus (MCPyV) genome (1, 2). Virus-positive MCC (MCCP) minimally expresses the viral oncoproteins large and small T antigen (LT and ST) that contribute to oncogenesis and the maintenance of MCC (3, 4). Similar to other polyomaviruses (5), MCPyV LT contains an LXCXE motif essential for binding and inhibiting the RB1 tumor suppressor to overcome the G_1_/S cell-cycle checkpoint (6). MCPyV ST induces the formation of a transcriptional coactivator complex containing MYCL and the EP400-Tip60 complex to activate expression of genes that contribute to MCC oncogenesis (7), including the p53-targeting E3 ubiquitin ligase *MDM2* (8). Since MCPyV LT and ST functionally disrupt the tumor suppressor activity of RB1 and p53, the *RB1* and *TP53* genes are often WT in MCCP (8–11). By contrast, virus-negative MCC (MCCN) has a highly elevated tumor mutational burden caused by excessive UV exposure that often includes inactivating mutations in *RB1* and *TP53* (9, 10, 12).

Both forms of MCC can express synaptophysin (SYP), chromogranin A (CHGA), and neurofilament (NEFH/M/L) typical for high-grade NE carcinomas (13, 14). In addition, MCC also shares transcriptional similarity with normal mechanosensory Merkel cells including the differential diagnostic marker cytokeratin 20 (KRT20, also known as CK20). MCC also expresses other markers of Merkel cells including cytokeratin 8 (KRT8) and the transcription factors SRY-box transcription factor 2 (SOX2) and atonal BHLH transcription factor 1 (ATOH1) (15–17), whose transcriptional activity is essential for survival of MCC cells (7). MCC tumors lacking SOX2 and ATOH1 have not, to our knowledge, been described, although variable ATOH1 expression levels have been reported (18), with the presence of integrated MCPyV correlated with increased expression in MCC cell lines (11, 19). Nonetheless, a complete picture of how viral status and NE gene expression may interact to define MCC subtypes has not been described to the best of our knowledge.

### YAP1 and WWTR1 are silenced and growth suppressive in other NE cancers.

The Hippo pathway is a signaling cascade composed of the mammalian STE20-like proteins 1 and 2 (MST1/2) and large tumor suppressor 1 and 2 (LATS1/2) kinases, which directly regulate Yes1-associated transcriptional regulator (YAP1, also referred to as YAP) and WW domain–containing transcription regulator 1 (WWTR1, also known as TAZ) (20). In response to growth-suppressive conditions, the MST1/2 and LATS1/2 kinases become sequentially activated, resulting in LATS1/2-dependent phosphorylation of YAP1 and WWTR1, thereby marking them for polyubiquitination and degradation or retention in the cytosol by 14-3-3 proteins (21). Under growth-promoting conditions, the MST1/2 and LATS1/2 kinases remain inactive, allowing YAP1 and WWTR1 to enter the nucleus and complex with the TEA domain transcription factors (TEAD1–4) (21). YAP1-TEAD and WWTR1-TEAD complexes promote cell-cycle–dependent gene expression (22, 23), as well as additional activities and targets (24, 25). 

Aberrant activation of YAP1 and WWTR1 has been reported for many solid tumor types including breast, gastric, and non–small cell lung cancer. In these cancers, YAP1 and WWTR1 expression is associated with increased drug resistance, metastasis, and poor outcomes (26, 27). By contrast, transcript-level silencing of *YAP1* and *WWTR1* has been reported in hematological cancers such as multiple myeloma (MM) (28, 29) and NE cancers such as medullary thyroid cancer (30–32), NE prostate cancer (33), and small cell lung cancer (SCLC) (34–37). Expression of YAP1 or WWTR1 in MM and SCLC cell lines with silenced *YAP1* and *WWTR1* at baseline led to decreased cell viability (28, 29, 37). Nonetheless a variant SCLC subtype has been described that expresses YAP1, albeit with reciprocal lower expression of the NE transcriptional regulator achaete-scute family BHLH transcription factor 1 (ASCL1) (34–36). Furthermore, SCLC exhibits intratumoral heterogeneity wherein ASCL1^hi^ or SYP^hi^ tumors contain small subpopulations of YAP1-expressing cells, suggesting transcriptional plasticity in the NE transcriptional program (38, 39). Another recent study showed YAP1-dependent proliferative loss in *YAP1*-silenced cancers (40), but this effect remains poorly studied in NE cancers other than SCLC.

Here, we found that MCC, like SCLC, could be distinguished by inversely correlated expression of YAP1 and WWTR1 and NE-associated genes and displayed intratumoral heterogeneity, in which NE^hi^ tumors contained a subpopulation of YAP1- and WWTR1-expressing cells. Moreover, we show that expression of YAP1 or WWTR1 in a NE^hi^ MCCP cell line could reduce cell viability and induce a cell-cycle arrest at least in part through TEAD-dependent transcriptional suppression of MCPyV LT. Our work supports a model in which silencing of YAP1 and WWTR1 is essential for tumorigenesis of MCPyV-positive MCC and highlights the growing concept of exclusivity between YAP1- and WWTR1- and NE-driven transcriptional programs.

## Results

### NE^hi^ and NE^lo^ transcriptional subtypes are observed at the single-cell and bulk levels in MCC tumor biopsies.

**Figure 1:**
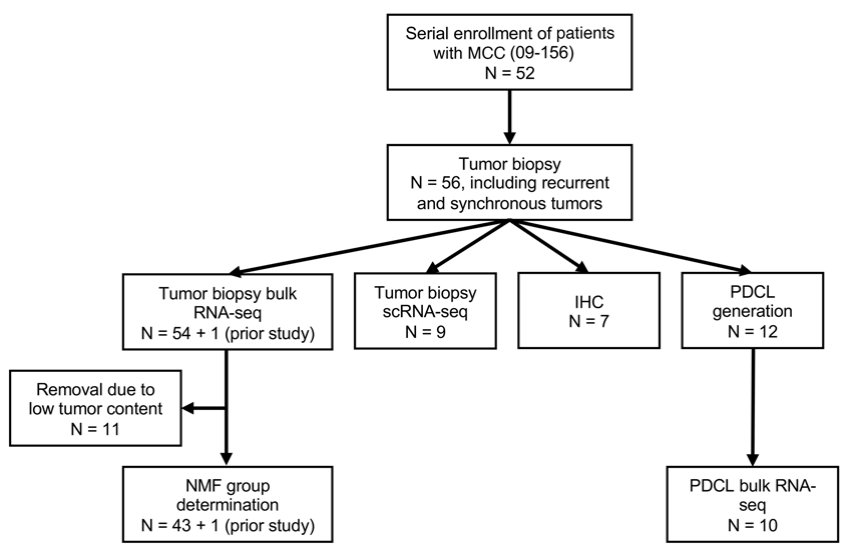
Flowchart of analyses of patient-derived MCC tumor biopsies performed in this study. Tumor biopsies from enrolled patients were subjected to bulk and scRNA-Seq, IHC, and the generation of PDCLs.

**Figure 2:**
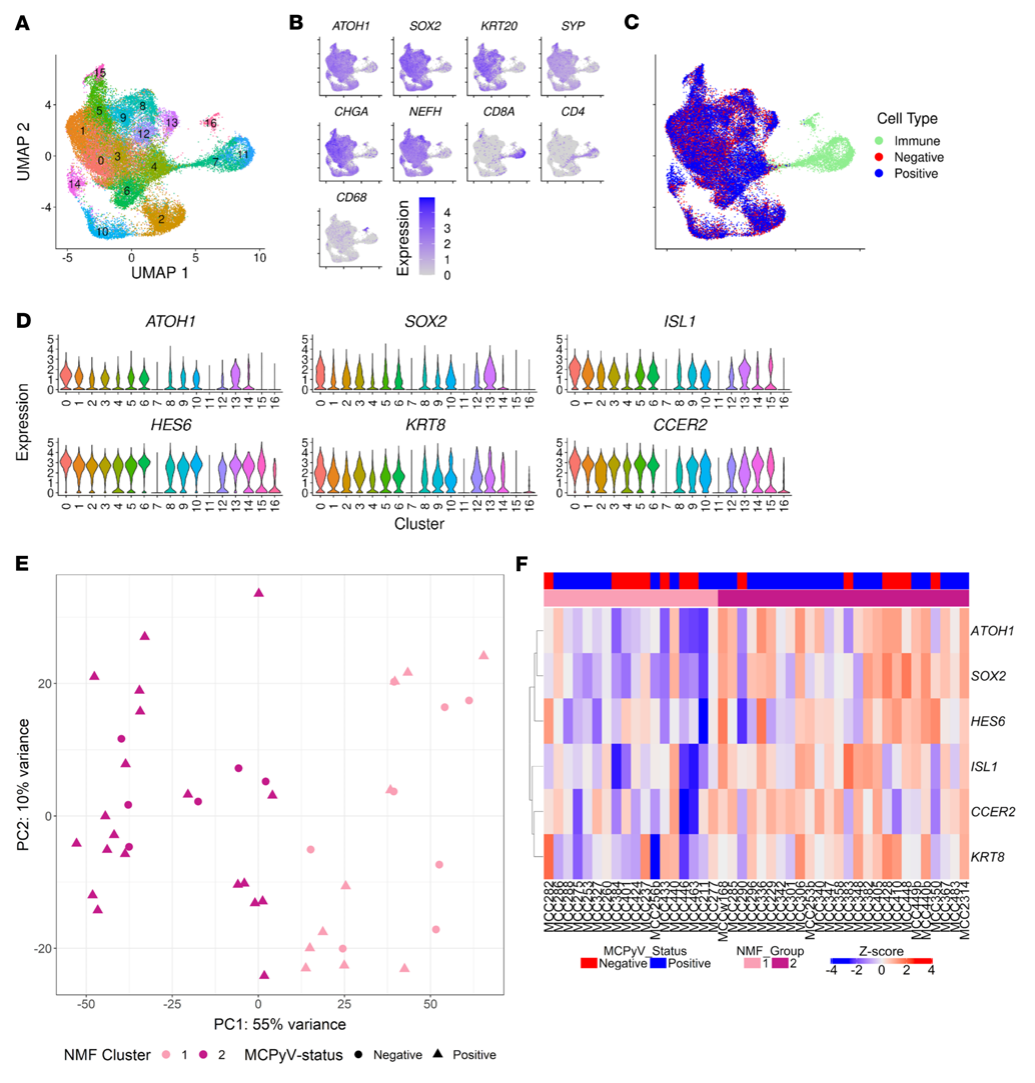
Bulk and scRNA-Seq of patient-derived MCC tumor biopsies identifies subgroups based on the expression of NE genes. (**A**) Dimensional reduction plot (DimPlot) of integrated scRNA-Seq analysis of all cells from 9 patient-derived tumor biopsies with clusters defined (resolution = 0.75). UMAP, uniform manifold approximation and projection. (**B**) Feature plots of MCC (*ATOH1*, *SOX2*, *KRT20*), NE (*SYP*, *CHGA*, *NEFH*), and immune cell markers (*CD8A*, *CD4*, *CD68*). (**C**) DimPlot as in **A**, but colored on the basis of MCPyV status of the tumor biopsy or cell presence in the immune cell clusters. (**D**) Violin plots comparing normalized expression of conserved cluster 0 genes between clusters. (**E**) PCA of bulk RNA-Seq from 44 patient-derived MCC tumor biopsies showing viral status (symbol shape) and NMF group (symbol color). (**F**) Expression of each cluster 0 gene in the 44 MCC tumor biopsies clustered by NMF group. MCC253b, MCC256b, and MCC334b represented recurrences of MCC253, MCC256, and MCC334 tumors, respectively. MCC440b represented a distinct primary tumor that presented synchronously with MCC440.

To assess the transcriptional environment present within MCC tumors, we performed a variety of analyses on biopsies obtained from patient tumors (Figure 1). First, we performed single-cell RNA-Seq (scRNA-Seq) on 9 patient-derived tumor biopsies to obtain 38,077 high-quality cells (Figure 2A). Of the 9 tumor samples, 6 were virus-positive, with 25,062 cells defined by non-zero expression of MCPyV *LT* or *ST* transcripts, and 3 were virus-negative, with 13,015 cells (Supplemental Figure 1A). All samples were integrated into a single clustering analysis using Seurat (41) to identify pan-MCC cellular subgroupings (Figure 2A). Seventeen unique transcriptional clusters were identified (resolution = 0.75), the majority of which (clusters 0–6, 8–10, and 12–15, representing 89.4% of all cells) were broadly categorized as MCC, as defined by the expression of *ATOH1*, *SOX2*, *KRT20*, and the NE markers *SYP*, *CHGA*, and *NEFH* (Figure 2B). In addition to MCC tumor cells, we observed clusters of immune cells including CD4-positive and CD8A-positive T cells (clusters 7 and 11) and CD68-positive macrophages (cluster 16). Every cluster was present within each tumor sample, although the relative proportions varied (Supplemental Figure 1B). Notably, the heterogeneity of MCC cell clustering was not due to viral status, as we observed coclustering of MCC cells from virus-positive and virus-negative tumors (Figure 2C). Thus, we wondered if factors other than MCPyV contributed to the observed transcriptional heterogeneity in MCC.

To assess transcriptional drivers of the different MCC clusters, we identified genes significantly enriched within a single cluster relative to all other clusters (Supplemental Table 1). We further assessed these cluster-enriched genes for conservation across all tumor specimens to identify high-confidence, cluster-specific enrichment (Supplemental Table 2). Cells in cluster 0 were enriched for 6 genes that included 3 markers of normal Merkel cells (*ATOH1*, *SOX2*, and *KRT8*), the Hes family BHLH transcription factor 6 (*HES6*), insulin gene enhancer protein (*ISL1*), previously shown to be highly expressed in MCC and other NE cancers (19, 42, 43), and coiled-coil glutamate-rich protein 2 (*CCER2*), not previously associated with MCC or NE transcriptional programs. As a result, we reasoned that cluster 0 represented the prototypic NE MCC cell population. Importantly, these cluster 0–enriched genes were broadly, although variably, expressed in all MCC, but not immune-associated, cell clusters (Figure 2D). These results suggest that the transcriptional heterogeneity within MCC may be based on or correlate with NE gene expression, rather than viral status.

To further explore transcriptional heterogeneity in MCC, we analyzed bulk RNA-Seq profiling of 55 patient-derived tumor biopsies (44) that included paired tissue samples for 7 of the 9 samples analyzed by scRNA-Seq (Supplemental Table 3). Viral status was determined by the expression of MCPyV *LT* and *ST* (Supplemental Figure 1C). Since tumor biopsies can contain normal skin or immune cells, we curated our data set for higher-purity samples on the basis of the expression of the cluster 0–enriched genes defined in the scRNA-Seq data (Supplemental Figure 1D). Eleven of the 55 samples had minimal expression of these as well as other MCC-specific and NE genes (Supplemental Figure 1E). Both Euclidean clustering (Supplemental Figure 1D) and principal component analysis (PCA) (Supplemental Figure 1F) showed divergence of these samples from the remaining 44, suggesting they contained a high proportion of nontumor cells. These samples were removed from further analysis. PCA of the top 500 most variably expressed genes (Supplemental Table 4) was used to assess the clustering of the 44 remaining samples (Figure 2E). Similar to the scRNA-Seq, we observed coclustering of virus-positive and virus-negative tumor samples. To identify transcriptional subgroupings within the bulk RNA-Seq data set, we performed non-negative matrix factorization (NMF) of the top 500 most variably expressed genes. We imputed a range of factorization ranks (*k*) to define the ideal value for subgroup decomposition (45), which was optimized at *k* = *2* (cophenetic correlation = 0.952), indicating 2 groups. These NMF-based transcriptional groups did not correlate strongly with viral status, with 76.9% (20 of 26) being MCPyV-positive in the group 2 samples compared with 55.6% (10 of 18) in group 1 (Figure 2E).

Since we observed variability in NE gene expression in the scRNA-Seq data, we wondered whether the NMF-defined groups may diverge based on expression of the NE cluster 0–enriched genes (Figure 2F). Generally, we found that NMF group 1 was associated with lower expression of cluster 0 genes, while group 2 was associated with higher expression, leading us to call them NE^lo^ and NE^hi^, respectively. However, a gradient of cluster 0 gene expression was apparent, suggesting that NMF groups 1 and 2 likely represent 2 overlapping transcriptional profiles or a spectrum of NE gene expression.

### YAP1, WWTR1, and NE marker expression is negatively correlated in MCC on a bulk and single-cell basis.

**Figure 3:**
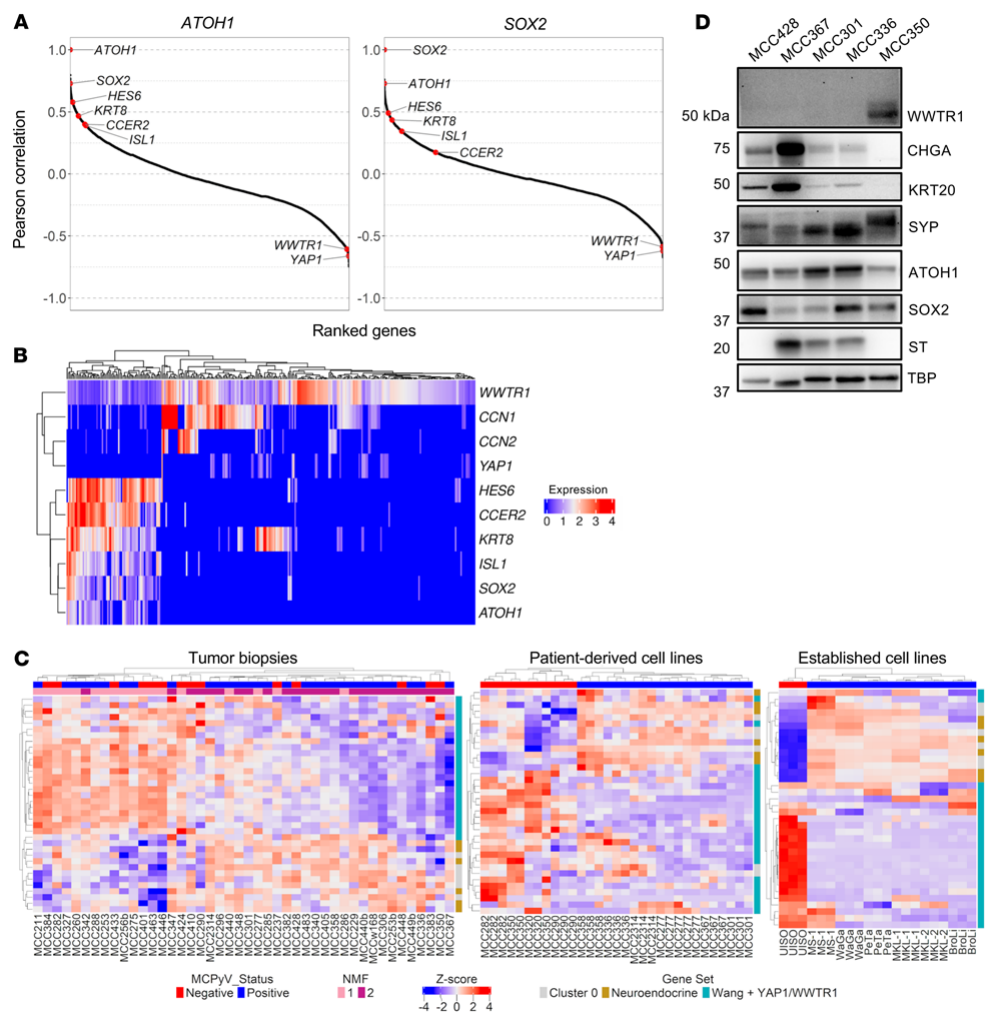
NE marker and YAP1 and WWTR1 expression is strongly negatively correlated in MCC tumor samples. (**A**) Genome-wide gene-to-gene Pearson correlations for 44 bulk RNA-sequenced tumor samples (44) showing that *YAP1* and *WWTR1* are negatively correlated with *ATOH1* and *SOX2* across all samples. (**B**) Heatmap of normalized expression of *YAP1*, *WWTR1*, *CCN1*, *CCN2*, and cluster 0 genes in each *WWTR1*-expressing cell (*n* = 301) from the 9 tumor samples analyzed by scRNA-Seq. (**C**) Heatmaps of the 44 bulk RNA-sequenced tumor samples (left) (44), bulk RNA-sequenced PDCLs with individual replicates shown (center) (44), and bulk RNA-sequenced, DMSO-treated established MCC cell lines (right) (11) showing negatively correlated expression between *YAP1* and *WWTR1* and their target genes (“Wang targets”) (47), cluster 0 genes, and other NE genes. (**D**) Immunoblot of PDCLs showing negatively correlated expression between WWTR1 and NE markers. *n* = 1.

Analysis of the scRNA-Seq and bulk RNA-Seq data sets revealed heterogeneity of NE gene expression within and across MCC tumors. We reasoned that genes whose expression was negatively correlated with the cluster 0 genes may help to define the NMF group 1 (the NE^lo^ group) tumor samples. Genome-wide, gene-to-gene Pearson correlations were performed on the 44-sample bulk RNA-Seq data set using the 6 cluster 0 genes (Figure 3A, Supplemental Figure 2A, and Supplemental Table 5). Among the most negatively correlated genes compared with *SOX2* and *ATOH1* were *YAP1* (*R^2^*: –0.62 and –0.66, respectively) and *WWTR1* (*R^2^*: –0.59 and –0.61, respectively). *YAP1* and *WWTR1* expression was also negatively correlated with expression of the other cluster 0 genes (Supplemental Figure 2A). Intriguingly, expression of YAP1 has been used to describe a non-NE subtype of SCLC (34–36, 39). IHC revealed a lack of YAP1 expression in MCC and other NE carcinomas with high expression of NE markers (46). As such, we postulated that some of the heterogeneity observed in the scRNA-Seq and bulk RNA-Seq data could be explained by the reciprocal expression of the NE cluster 0 genes relative to non-NE-associated *YAP1* and *WWTR1*.

Reassessment of the scRNA-Seq data set revealed a small population of cells in clusters 8 and 12 that expressed *YAP1*, *WWTR1*, and their direct transcriptional targets cellular communication network factor 1 (*CCN1*, also known as *CYR61*) and cellular communication network factor 2 (*CCN2*, also known as *CTGF*) (Supplemental Figure 2B). Indeed, the less conservative cluster enrichment analysis identified *WWTR1* as being significantly enriched in cluster 12 (Supplemental Table 1). *WWTR1*-expressing cells were rare but present in all samples, albeit at different proportions, and *YAP1*-expressing cells were present in 8 of 9 samples (Supplemental Figure 2, C and D). *YAP1-* and *WWTR1-*expressing cells were not restricted to either MCCP or MCCN tumors or either NMF group, as assayed by bulk RNA-Seq. In general, *WWTR1* was more strongly expressed and in more cells per sample compared with *YAP1* across all samples (Supplemental Figure 2D). Extraction of all *WWTR1*-positive cells revealed a clear negative correlation between expression of the NE cluster 0 genes and *YAP1*- and *WWTR1*-associated genes (Figure 3B). Importantly, we observed low levels of *WWTR1* in single cells with high expression of the cluster 0 markers (Figure 3B, left), indicating that *WWTR1* expression was present in clearly identifiable MCC cells, albeit at reciprocally lower levels. Furthermore, we also found *KRT8*, a marker of normal Merkel cells and MCC, coexpressed in single cells with higher levels of *WWTR1* (Figure 3B, center) indicating that these *WWTR1*-expressing cells probably represented MCC tumor cells. Taken together, these results indicate that *YAP1* and *WWTR1* expression could be observed in bona fide MCC at the single-cell level and was generally negatively correlated with NE gene expression.

IHC was performed on a panel of MCC tumors to determine whether subpopulations of YAP1- and WWTR1-expressing cells could be observed in situ. Consistent with previous reports (14, 19), all H&E-positive tumor sections stained for ATOH1 (7 of 7) and most for KRT20 (5 of 7) (Supplemental Figure 3 and Supplemental 4A). Strikingly, expression of ATOH1 and KRT20 varied in intensity and positivity within individual tumor sections (Supplemental Figure 4, B and C), indicating the potential for heterogenous subpopulations. Staining for YAP1 and WWTR1 revealed a few rare cells that were positive within the tumor sections assessed. We observed strong focal staining of YAP1 and WWTR1 in a subpopulation of cells within a tumor section that was otherwise positive for H&E and ATOH1 (Supplemental Figure 5, red arrows). Importantly, the staining of WWTR1-positive and YAP1-positive cells within this region was nuclear, suggesting the potential for WWTR1/YAP1-mediated transcriptional activity within these cells.

We asked whether the reciprocal expression of *YAP1*- and *WWTR1-*dependent genes and NE markers could be observed in the bulk RNA-Seq of the 44 tumor biopsies. We plotted the expression of the NE cluster 0 genes plus the MCC-specific marker *KRT20* and additional NE markers including *CHGA*, *SYP*, and *NEFH/M/L*, as well as *YAP1*, *WWTR1*, and a curated list of 21 known direct transcriptional targets of YAP1 and WWTR1 (Figure 3C, left) (47). Not only was the negative correlation between the cluster 0/NE markers and *YAP1*- and *WWTR1*-associated genes clearly observed, but there was also good agreement with the NMF grouping.

In addition to the 44 tumor biopsies, we observed reciprocal expression of the YAP1- and WWTR1-associated and cluster 0/NE gene sets in transcriptional profiling (44) of patient-derived cell lines (PDCLs) (Figure 3C, center; Supplemental Table 6). There was general concordance between a PDCL’s transcriptional pattern and its associated tumor’s NMF grouping, with 5 of 6 NE^hi^ PDCLs derived from group 2 tumor samples and 1 of 3 NE^lo^ PDCLs from a group 1 tumor sample. Two of the PDCLs that differed from their associated tumor sample in classification were MCC290 and MCC350, which clustered with the NE^lo^ cell lines despite having associated group 2 tumor samples. Differences in expression pattern may reflect cell lines derived from subpopulations of YAP1- and WWTR1-expressing cells within the tumor or from culture conditions.

Quantitative reverse transcription PCR (RT-qPCR) analysis of *YAP1* and *WWTR1* in 3 PDCLs confirmed the RNA-Seq findings (Supplemental Figure 6A). Immunoblot analysis showed that WWTR1 expression was detectable in the MCCN PDCLs MCC350 (Figure 3D) and MCC410 (Supplemental Figure 6B). Notably, the MCC350 and MCC410 PDCLs had reciprocally lower expression of NE markers, including CHGA and ATOH1, compared with the other PDCLs (Figure 3D and Supplemental Figure 6B). YAP1 was not detectable in any of the PDCLs. We noted that the PDCL RNA-Seq displayed a stronger relationship between transcriptional status and viral status than did the tumor biopsy RNA-Seq, with only MCCN lines being classified as NE^lo^. However, not all MCCN cell lines expressed WWTR1 protein, as exemplified by MCC428 (Figure 3D).

We also examined previously published RNA-Seq data sets (11) of the established MCC cell lines MKL-1, MKL-2, MS-1, WaGa, PeTa, BroLi, and UISO treated with DMSO (Figure 3C, right). Again, we observed a negative correlation between expression of the YAP1- and WWTR1-associated genes and the cluster 0/NE genes in this data set. We performed RT-qPCR and immunoblotting (Supplemental Figure 6, C and D) to confirm these findings for a subset of the cell lines, as well as in the virus-negative lines MCC13 and MCC26. In contrast to the PDCLs, we detected YAP1 protein in the 3 WWTR1-positive MCCN established cell lines. The reciprocal expression pattern between YAP1 and WWTR1 and the cluster 0/NE genes and the correlation with viral status was more evident in the established cell line RNA-Seq data than it was for the PDCLs or 44 tumor biopsies. Of note, the core Hippo pathway kinases were expressed in all the established cell lines (Supplemental Figure 6E) and PDCLs (Supplemental Figure 6B), including in ones that did not express YAP1 or WWTR1, and most cell lines had some degree of MOB1 phosphorylation, an indicator of Hippo pathway functionality and activation (48).

### YAP1, WWTR1, and NE marker expression correlates with morphology of MCC cell lines.

**Figure 4:**
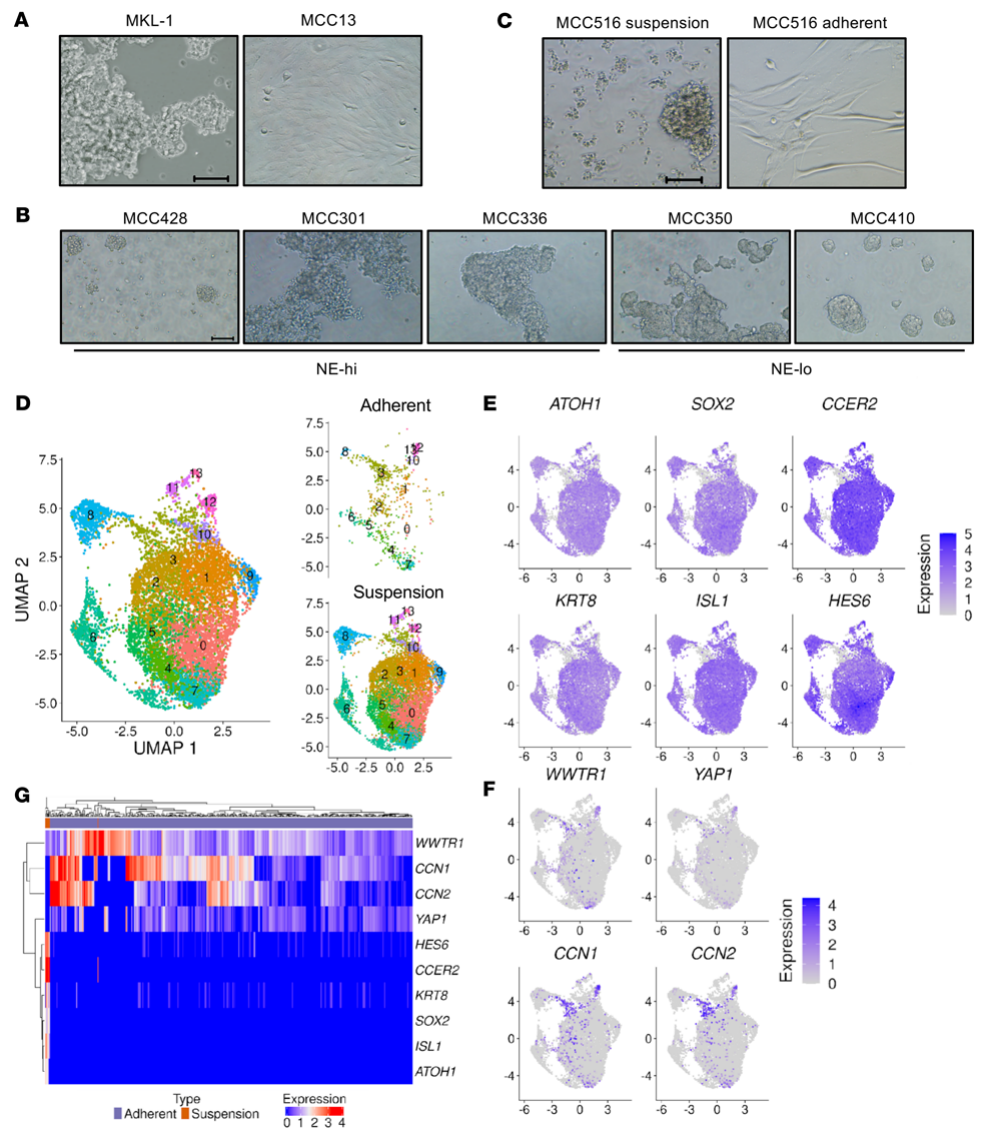
YAP1, WWTR1, and NE gene expression correlates with MCC cell line morphology. (**A**) Morphologic comparison of MKL-1 and MCC13 established cell lines. (**B**) Morphologic comparison showing that NE^lo^ PDCLs exhibited increased clumping versus NE^hi^ cell lines. (**C**) Morphologic comparison between divergent populations of the PDCL MCC516. (**A**–**C** ) Original magnification, ×10. Scale bars: 100 μm. *n* = 1 for each image. (**D**) DimPlots of integrated scRNA-Seq clustering analysis of 2 divergent suspension (MCC516s) and adherent (MCC516a) PDCLs derived from the same parent population. (**E**) Feature plots of normalized expression of cluster 0 genes and (**F**) *YAP1*, *WWTR1*, *CCN1*, and *CCN2* expression in MCC516s/a cells. (**G**) Heatmap of normalized expression of *YAP1*, *WWTR1*, *CCN1*, *CCN2*, and cluster 0 genes in each *WWTR1*-expressing cell (*n* = 446) from the MCC516s/a samples analyzed by scRNA-Seq.

We noticed the transcriptional profiles of both the PDCLs and established cell lines were strongly correlated with morphology. The established cell lines were clearly distinguished, with the NE^hi^ lines growing as suspended neurospheres, while the NE^lo^ lines were adherent (Figure 4A). Although all PDCLs formed neurospheres, they displayed a spectrum of density and adhesiveness. The NE^hi^ lines MCC428, MCC301, and MCC336 formed loose and chain-like or sheet-like clumps that were easily dissociated by gentle pipetting, whereas the NE^lo^ lines MCC350 and MCC410 formed the densest and most difficult-to-dissociate clumps (Figure 4B).

Morphological plasticity was also observed in some of the PDCLs. Upon enzymatic dissociation during routine passaging, an adherent population of cells developed from suspended MCC516 neurospheres. We separately cultured the adherent (MCC516a) and suspension (MCC516s) cell populations for 9 weeks and then harvested both populations for scRNA-Seq analysis (Figure 4C). The scRNA-Seq resulted in 8,368 high-quality cells from MCC516s cells and 782 from MCC516a cells. The data were integrated into a single clustering analysis to identify conserved transcriptional groupings between culturing conditions. We identified 13 clusters, most of which expressed the cluster 0 genes (Figure 4, D and E). Importantly, we observed coclustering transcriptional populations of MCC516a and MCC516s cells (Figure 4D). Furthermore, both MCC516a and MCC516s lines contained populations of cells expressing *MKI67*, a critical marker of proliferation (49), consistent with actively growing cells in both culturing methods (Supplemental Figure 7A). Expression of *YAP1*, *WWTR1*, *CCN1*, and *CCN2* was detected within clusters 3 and 12, and these clusters were enriched for adherent cells relative to the suspension culture (Figure 4F and Supplemental Figure 7B). Extraction of all *WWTR1*-positive cells again showed a negative correlation between *WWTR1/YAP1* and NE/cluster 0 gene expression (Figure 4G). Importantly, the *WWTR1*-positive cells were nearly exclusively adherent. However, we observed *KRT8-*expressing *WWTR1*-positive cells in the adherent cells (Figure 4G), indicating that these non-NE cells were probably MCC. A small number of *WWTR1*-positive cells were also present in the NE^hi^ MCC516s population (Figure 4G), albeit with reciprocally lower expression of *WWTR1*, identifying a potential source population for MCC516a cells.

### NE^lo^ MCC trends with recurrent disease.

**Figure 5:**
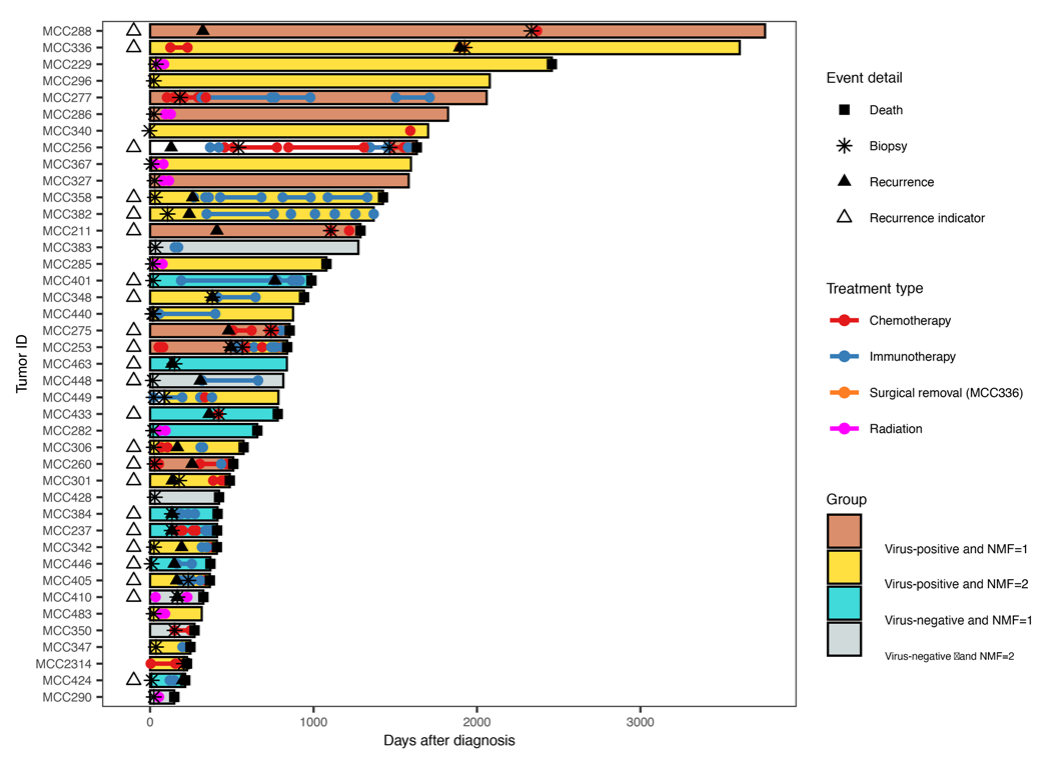
NE^lo^ MCC may be associated with recurrent disease. Swimmer plot of selected metadata for 41 patients with tumor biopsies categorized by NMF- and MCPyV-defined subgroupings. White column sections indicate that the sample was removed from the tumor biopsy RNA-Seq analyses due to an insufficient tumor sample. Three tumor biopsies present in the bulk RNA-Seq represented recurrences of tumors from the same patient (MCC253b, MCC256b, MCC334b), and a single patient had synchronous primary tumors (MCC440b). With respect to NMF grouping in this analysis, we only considered the first viable tumor biopsy as representative for the patient. White triangles indicate patients for whom recurrence was observed, while black triangles within bars indicate the timing of recurrence.

We examined patient metadata associated with the tumor biopsies to determine the clinical relevance of the NE^hi^ and NE^lo^ transcriptional groups (Table 1 and Figure 5). NE^hi^ and NE^lo^ transcriptional groups did not differ significantly in terms of overall survival (OS) or recurrence-free survival (RFS) (Supplemental Figure 8, A and B). However, classifying tumors on the basis of viral status revealed that patients with MCCN had significantly reduced OS and RFS compared with those with MCCP (Supplemental Figure 9, A and B), consistent with prior observations (50). Combining the 2 classification schemes revealed clustering of patients in terms of OS and RFS within virus-defined subgroupings independent of the NE-based transcriptional subtypes (Supplemental Figure 10, A and B). YAP1 expression has been associated with recurrent disease in prostate cancer (51) and drug-resistant disease in hepatocellular, ovarian, pancreatic, and breast cancers (52). Interestingly, 72.2% (13 of 18) of patients with NE^lo^ MCC, as determined by the tumor biopsy RNA-Seq, developed recurrent disease as compared with only 43.5% (10 of 23) of patients with NE^hi^ disease (Table 1 and Figure 5). The recurrence rate of NE^lo^ MCC was much higher than a previously reported study, which found an overall 5-year recurrence rate of 40% for MCC (53). However, given the limited sample size, these results are descriptive and not necessarily predictive.

### YAP1 and WWTR1 expression in NE^hi^ MCCP cells induces TEAD-dependent growth-suppressive transcriptional changes.

**Figure 6:**
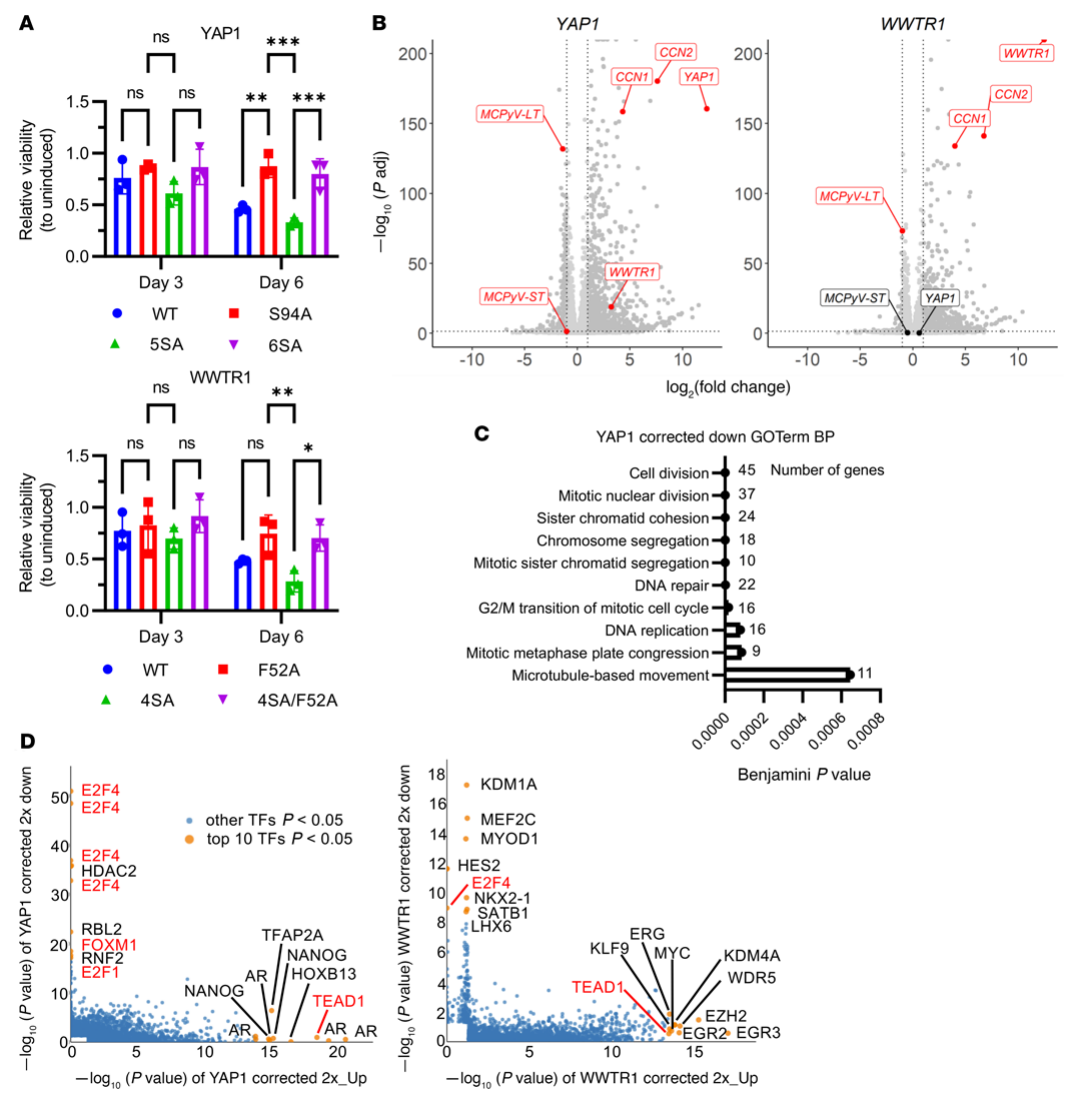
Expression of YAP1 or WWTR1 in NE^hi^ MCCP cells causes growth-suppressive, TEAD-dependent transcriptional changes. (**A**) CellTiter-Glo viability assay of MKL-1 cells inducibly expressing WT or mutant YAP1 or WWTR1 for 3 and 6 days and compared with uninduced cells for each expression line. *n* = 3. Data represent the mean ± SEM. *P < 0.05, **P < 0.01, and ***P < 0.001, by 2-way, repeated-measures ANOVA with Tukey’s post hoc test for each gene. (**B**) Volcano plots highlighting DEGs (red) in YAP1 or WWTR1 verses GFP conditions. Vertical dotted lines indicate a log_2_ fold change of greater than 1 or of less than –1. Horizontal dotted line indicates a *P*adj of less than 0.05. (**C**) GO term biological process (BP) analysis of 2-fold downregulated (2×_Down) DEGs in the corrected YAP1 data set. (**D**) LISA of corrected 2-fold upregulated (2×_Up) and 2-fold downregulated DEGs in the YAP1- and WWTR1-expressing conditions.

Expression of YAP1 and WWTR1 in *YAP1*- and *WWTR1*-silenced cancers, including NE^hi^ SCLC, can suppress proliferation (28, 29, 37, 40). To determine whether expression of YAP1 and WWTR1 could also suppress proliferation in NE^hi^ MCCP, we transduced MKL-1 cells with doxycycline-inducible (DOX-inducible) WT or mutant YAP1 or WWTR1. The YAP1 and WWTR1 mutants included transcriptionally hyperactive YAP1 5SA (54) and WWTR1 4SA (55), TEAD-binding–defective mutants YAP1 S94A (56) and WWTR1 F52A (57, 58), and constructs combining both types of mutations, YAP1 6SA and WWTR1 4SA/F52A. Upon induction, the WT and hyperactive, but not TEAD-binding–defective or dual-mutant constructs, increased expression of the YAP1 and WWTR1 direct transcriptional target CCN1 (Supplemental Figure 11A and Supplemental Figure 12A). Expression of WT or hyperactive YAP1 significantly decreased cellular viability after 6 days of induction as compared with expression of TEAD-binding–defective or dual-mutant YAP1 (Figure 6A, upper panel). In the case of WWTR1, only induction of the hyperactive and not the WT protein significantly reduced cellular viability at the 6-day time point as compared with TEAD-binding–defective or dual-mutant WWTR1 (Figure 6A, lower panel). Interestingly, expression of WT or hyperactive YAP1 or WWTR1, but not TEAD-binding–defective or dual-mutants, led to the formation of dense, difficult-to-dissociate neurospheres that were similar in appearance to WWTR1-expressing PDCL MCC410 neurospheres (Figure 3B, Supplemental Figure 11B, and Supplemental Figure 12B).

Since induction of transcriptionally active YAP1 or WWTR1 reduced proliferation in NE^hi^ MCCP, we performed transcriptome profiling after 3 days of treatment (prior to the onset of reduced viability) to identify potential downstream effectors (Supplemental Table 7). As expected, previously published direct YAP1 and WWTR1 target genes (47) were broadly induced in the WT but not the TEAD-binding–defective mutant samples (Supplemental Figure 13A). We defined differentially expressed genes (DEGs) as those with an absolute log_2_ fold change cutoff of 1 or higher and an adjusted *P* value (*P*adj) of 0.05 or less, compared with levels in GFP-expressing control cells (Supplemental Tables 8–11). The DEGs from WT YAP1 and WWTR1 were subtracted from the DEGs of their respective TEAD-binding–defective mutants to identify genes specifically transcriptionally regulated by YAP1-TEAD and WWTR1-TEAD complexes (“corrected targets;” Supplemental Figure 13B and Supplemental Tables 12 and 13). Strikingly, among the most significantly downregulated genes in both the corrected YAP1 and WWTR1 targets was MCPyV *LT*; MCPyV *ST* was also downregulated. In line with this finding, MCPyV *LT* was significantly downregulated in both YAP1- and WWTR1-expressing cells compared with GFP conditions (Figure 6B). Since MCPyV LT can overcome RB1-dependent G_1_/S cell-cycle arrest in MCCP cell lines (6), we reasoned that LT downregulation could contribute to the YAP1- and WWTR1-mediated proliferative defect. We performed RT-qPCR and observed a roughly 2-fold, although not statistically significant, downregulation of MCPyV *LT* and *ST* transcripts upon YAP1 or WWTR1 induction, concordant with the RNA-Seq results (Supplemental Figure 13C). Interestingly, expression of transcriptionally inactive YAP1 or WWTR1 led to increased MCPyV *LT* and *ST* transcript levels. To identify gene sets significantly enriched or depleted upon YAP1 and WWTR1 induction, we used the Database for Annotation, Visualization, and Integrated Discovery (DAVID) (59, 60) to perform a gene ontology (GO) term analysis of the corrected DEGs (Figure 6C and Supplemental 13, D and E). Upregulated GO terms for both YAP1 and WWTR1 were related to extracellular matrix organization and cell adhesion, concordant with the morphological changes we observed upon YAP1 and WWTR1 expression (Supplemental Figures 11 and 12, Supplemental Figure 13, D and E, and Supplemental Table 14). These GO terms included several integrin and collagen subunits that were significantly upregulated in our transcriptional profiling (Supplemental Figure 13F). Interestingly, we found that genes transcriptionally downregulated by YAP1 were significantly enriched for GO terms involving DNA replication, chromosome segregation, and, more broadly, cell division (Figure 6C). We did not observe a significant enrichment for cell-cycle GO terms in the WWTR1 downregulated DEGs, in line with WWTR1’s more moderate effect on cellular viability and transcription (Figure 6, A and B). To predict transcription factors that may directly regulate these genes based on publicly available ChIP-Seq data sets, we performed landscape in silico deletion analysis (LISA) (61) on the corrected up- and downregulated DEGs (Figure 6D). As expected, TEAD1 was one of the most significant hits for the corrected YAP1 and WWTR1 upregulated gene lists. For the corrected downregulated YAP1 and WWTR1 targets, we identified several members of the cell-cycle–regulating E2F family of transcription factors (62). We also observed targets of another cell-cycle–regulating transcription factor, FOXM1 (63), exclusively in the YAP1 downregulated targets. Together, these data suggest that TEAD-dependent transcriptional repression of MCPyV *LT* upon induction of YAP1 and WWTR1 in NE^hi^ MCCP may lead to RB1-dependent G_1_/S arrest and ultimately contribute to reduced cellular viability.

### Expression of YAP1 suppresses cell-cycle progression in NE^hi^ MCCP cells in part through indirect downregulation of MCPyV LT expression.

**Figure 7:**
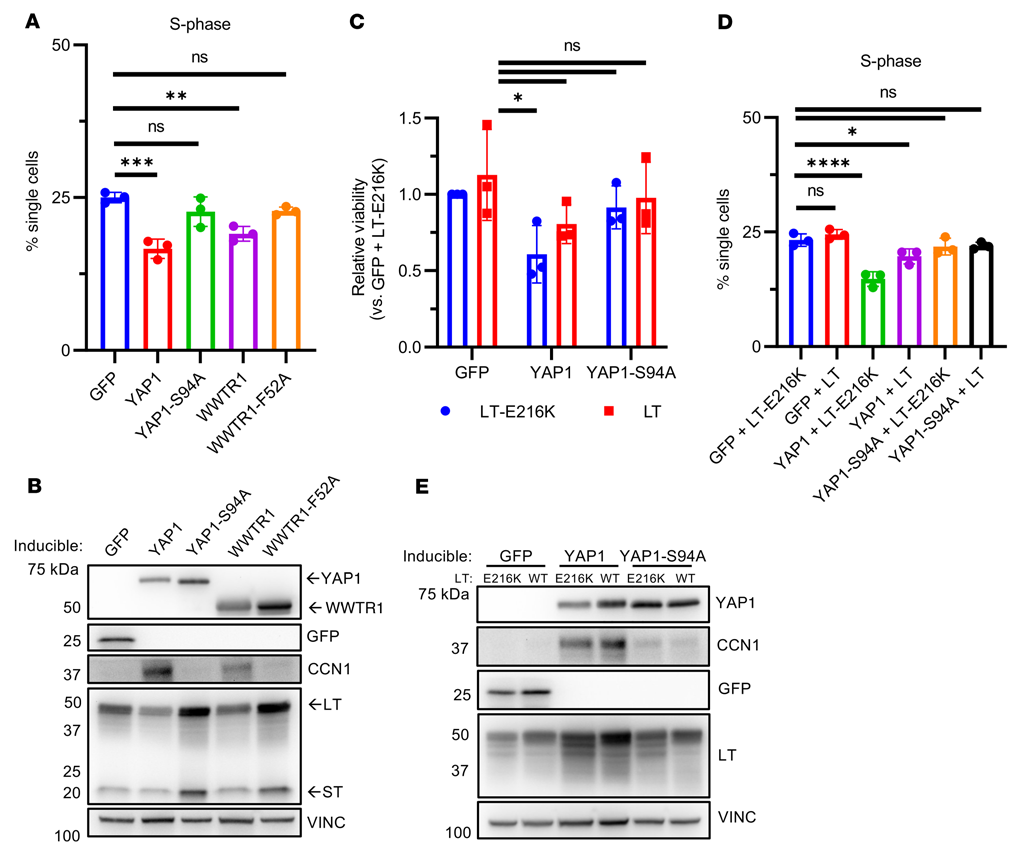
Induction of YAP1 expression enforces TEAD-dependent G_1_/S arrest through depletion of MCPyV LT. (**A**) Quantification of single cells present in the S-phase population from cell-cycle analyses of MKL-1 cells induced to express YAP1, WWTR1, or GFP constructs for 6 days. *n* = 3. Data represent the mean ± SD. **P < 0.01 and ***P < 0.001, by 1-way ANOVA with Dunnett’s post hoc test. (**B**) Immunoblot of cell lysates from the cells in **A**. Results are representative of 3 biological replicates. (**C**) MKL-1 cells with inducible WT or mutant YAP1 and constitutive expression of WT or RB1-binding–defective MCPyV LT (E216K) cells were induced for 6 days and assessed for viability via CellTiter-Glo. *n* = 3. Data represent the mean ± SEM. *P < 0.05, by 1-way ANOVA with Dunnett’s post hoc test. (**D**) Quantification of single cells present in the S-phase population from cell-cycle analyses of cells treated as in **C**. *n* = 3. Data represent the mean ± SD. *P < 0.05 and ****P < 0.0001, by 1-way ANOVA with Dunnett’s post hoc test. (**E**) Immunoblot of cognate cell lysates from **D**. L21 MCPyV LT is approximately the same molecular weight as MKL-1 endogenous LT. Results are representative of 3 biological replicates. VINC, vinculin.

We explored whether induction of YAP1 and WWTR1 in NE^hi^ MCCP MKL-1 cells induces cell-cycle arrest through the suppression of MCPyV LT expression. We found that induction of WT, but not TEAD-binding–defective, YAP1 or WWTR1 significantly decreased the percentage of cells in S phase (Figure 7A), while increasing those in G_1_ (Supplemental Figure 14A). No effect on G_2_/M cells was noted (Supplemental Figure 14A). Immunoblot analysis confirmed downregulation of MCPyV LT protein levels upon induction of transcriptionally competent YAP1 or WWTR1 (Figure 7B). We observed similar effects in a second NE^hi^ MCCP cell line, WaGa (Supplemental Figure 14B). These data support the notion that suppression of MCPyV LT protein levels in YAP1- or WWTR1-expressing MCCP cells may contribute to RB1-dependent cell-cycle dysregulation. In line with our RNA-Seq results, we observed an increase in MCPyV LT protein levels upon expression of TEAD-binding–defective YAP1 or WWTR1 mutants in MKL-1 cells (Figure 7B) and ST levels upon expression of TEAD-binding–defective YAP1 in WaGa cells (Supplemental Figure 14B).

To directly assess the requirement of LT-dependent RB1 suppression for YAP1-mediated cell-cycle dysregulation, we transduced MKL-1 cells containing inducible WT or TEAD-binding–defective YAP1 with a constitutive expression construct encoding MCPyV LT (L21) (64) or MCPyV LT with a mutated LXCXE motif (E216K) previously reported to abrogate RB1 binding (65). Constitutive expression of WT, but not RB1-binding–defective MCPyV LT, led to a partial rescue of both cellular viability (Figure 7C) and normal S- and G_1_-phase populations (Figure 7D and Supplemental Figure 14C) in the context of YAP1 expression. However, constitutive expression of either WT or mutant MCPyV LT did not alter the appearance of the TEAD-dependent morphological changes observed upon YAP1 expression (Supplemental Figure 14D). Immunoblot analysis verified that MCPyV LT levels were not suppressed in the constitutively expressing cells, and CCN1 levels were increased following YAP1 induction (Figure 7E). These data support the idea that TEAD-dependent loss of MCPyV LT protein levels may contribute to RB1-dependent cell-cycle dysregulation upon YAP1 expression in NE^hi^ MCCP.

Despite the TEAD-dependent depletion of MCPyV LT levels by YAP1 and WWTR1, it was unclear if these effects were due to direct regulation of the integrated MCPyV genome by the YAP1-TEAD or WWTR1-TEAD transcriptional complexes. The related polyomavirus simian virus 40 (SV40) contains a consensus TEAD-binding motif (5′-GGAATG-3′) in the noncoding control region (NCCR), which has been shown to directly bind TEAD-containing transcriptional complexes (66, 67). We assessed the MCPyV (RefSeq: NC_010277.2) and SV40 (RefSeq: NC_001669.1) NCCRs for potential YAP1-TEAD– or WWTR1-TEAD–binding motifs (Supplemental Figure 14E). As expected, the TEAD-binding consensus motif was present in the NCCR of the SV40 genome, but the MCPyV NCCR lacked a TEAD-binding motif, indicating that YAP1 and WWTR1 are unlikely to directly bind and regulate the expression of MCPyV T antigens. Thus, the effects of YAP1 and WWTR1 on MCPyV LT protein and mRNA levels are probably indirect.

## Discussion

MCC is an aggressive NE carcinoma of the skin currently distinguished by 2 etiological subtypes defined by clonally integrated MCPyV or UV-induced mutagenesis. Despite these profound genetic differences, diagnostic IHC markers other than the expression of MCPyV LT do not readily distinguish between these subtypes (68, 69). Prior work has shown variable expression of NE genes in MCC (19), whereas studies of other NE cancers including SCLC have used NE transcription factors as a means of subtype deconvolution (34–38, 40). As such, we wondered if additional MCC subtypes could be identified thorough analysis of transcriptional data sets. Here, we characterized the expression profiles of MCC tumor biopsies, PDCLs, and established cell lines to identify a spectrum of NE gene expression that is negatively correlated with the expression of YAP1 and WWTR1. Our major findings indicated that (a) MCCP and MCCN transcriptional profiles broadly overlap and show considerable heterogeneity; (b) MCC can be classified according to bulk and single-cell-level reciprocal expression of NE markers and YAP1 and WWTR1; (c) a spectrum of NE and YAP1 and WWTR1 gene expression is present within MCC, independent of viral status; (d) NE^hi^ tumors can contain subpopulations of YAP1- and WWTR1-expressing cells that can subsequently be subcultured; (e) YAP1 and WWTR1 expression levels are strongly correlated with MCC cell morphology and influence cell-cell adhesiveness; and (f) YAP1 and WWTR1 expression in NE^hi^ MCCP cells inhibits cell-cycle progression, at least in part through indirect suppression of MCPyV LT.

Our finding that the cluster 0/NE markers and YAP1 and WWTR1 expression–based classification scheme was strongly correlated with cell morphology contextualizes earlier reports classifying MCC cell lines as either “classic” or “variant.” Classic MCC has been characterized by higher expression of NE genes and growth in suspension and variant MCC by lower NE gene expression and adherent growth (70–72). However, prior studies have cast doubt on how well the variant cell lines represented MCC tumors (19, 73). Our findings explain these observations through reclassification of the classic and variant cell lines into NE^hi^ and NE^lo^ subgroups, respectively. In general, other *YAP1*- and *WWTR1*-silenced cancers typically grow in suspension, while YAP1- and WWTR1-expressing cancers grow adherently (28, 29, 34, 37). Furthermore, we observed that expression of YAP1 and WWTR1 in a *YAP1*- and *WWTR1*-silenced NE^hi^ suspension cell line induced the formation of dense, difficult-to-dissociate neurospheres concurrent with the induction of integrin and collagen subunits that could increase adhesiveness. We observed correlated morphological and transcriptional plasticity in real time upon isolation of an adherent MCC PDCL derived from an otherwise suspended culture. Importantly, scRNA-Seq analysis revealed that the adherent culture had higher levels of YAP1 and WWTR1 with reciprocally lower NE gene expression than did the suspended culture. Taken together, our work supports a model in which the established MCC cell lines represent a spectrum of NE gene expression and that YAP1 and WWTR1 are key determinants of cell line adherent or suspension morphology. Our data suggest that the variant MCC cell lines may represent genuine transcriptional states identifiable within MCC tumors and may therefore be more physiologically relevant than previously appreciated.

Our findings are in concordance with prior work in SCLC, which has shown that cell lines fall into suspension and adherent subtypes corresponding with a NE and non-NE transcriptional profile, respectively (34). A more detailed classification scheme, based on transcription factor expression, has been proposed for SCLC. NE SCLC cells can be specified by expression of the NE-associated transcription factor ASCL1 or NEUROD1, whereas non-NE cells are specified by expression of the chemosensory-associated transcription factor POU2F3 or YAP1 (74). Similar to our scRNA-Seq findings in MCC, Ireland et al. (38) also found evidence for subpopulations of ASCL1^lo^YAP1^hi^ cells within otherwise ASCL1^hi^ SCLC tumors. Other reports have suggested that non-NE subpopulations such as these may be important for promoting metastasis (75) and drug resistance (52). In line with these ideas, we observed a modest, although nonsignificant, enrichment of recurrent disease in patients with tumor biopsies classified as NE^lo^. Nevertheless, all MCC expression profiles indicate that ATOH1 is an important lineage-specific transcription factor with little evidence for a role of ASCL1 or NEUROD1.

Prior work has shown that expression of YAP1 or WWTR1 is detrimental to the viability of NE^hi^ cancer cell lines (37, 40). We observed that expression of either YAP1 or WWTR1 led to decreased proliferation of NE^hi^ MCCP cells in a TEAD-dependent manner. Transcriptional profiling indicated that YAP1 and WWTR1 expression led to general downregulation of cell-cycle–associated genes and MCPyV LT. In concordance with the role of MCPyV *LT* in inhibiting the activity of RB1 to prevent G_1_/S arrest (6), E2F target genes were downregulated, and NE^hi^ MCCP cells dropped out of S phase into G_1_ upon induction of YAP1 or WWTR1. We noted that YAP1 expression tended to have a more severe growth-suppressive phenotype than did WWTR1 and was more effective at TEAD-dependently affecting gene expression. We speculate that this may reflect why some MCC tumors and PDCLs expressed WWTR1 and not YAP1; WWTR1 may be less antagonistic than YAP1 to the NE transcriptional program.

Altogether, these findings suggest that MCCP may, in particular, tend toward decreased levels of YAP1 and WWTR1 due to the requirement of MCPyV T antigen expression. Indeed, we found that the NE^hi^ NMF group 2 tumor biopsies were enriched for MCCP (76.9%, 20 of 26), whereas the NE^lo^ NMF group 1 biopsies were split more evenly (55.6% MCCP, 10 of 18). We speculate that the apparent need for a NE^hi^ state to maintain MCPyV T antigen expression may hold clues to the cell of origin of MCC. Given the ability of YAP1 and WWTR1 to repress MCPyV T antigen expression, we propose that only cells with low or absent expression of YAP1 and WWTR1 will support MCCP oncogenesis. With regard to MCCN, our patient metadata were consistent with prior findings that MCCN has a poor prognosis compared with MCCP and suggested that the NE^lo^ group is associated with a trend toward increased disease recurrence (50). Given the relatively higher proportion of MCCN tumors in the NE^lo^ NMF group 1, we speculate that YAP1 or WWTR1 expression could contribute to the poor prognosis for patients with MCCN, especially in light of the previously documented roles of YAP1 and WWTR1 in recurrence and drug resistance in other cancers (51, 52).

Despite our work demonstrating that YAP1 overexpression transcriptionally suppressed MCPyV T antigen levels, prior work has shown that MCPyV ST overexpression can enhance YAP1 transcriptional activity through ST-mediated inhibition of protein phosphatase 2A (PP2A) (76). Although these effects may be valid, this study was performed in primary non-NE human foreskin fibroblasts (BJ) and human mammary epithelial cells (HMECs), both of which expressed high levels of YAP1 protein at baseline. As a result, the indirect interaction between ST and YAP1 in the context of a non-NE transcriptional state and overexpression may differ from the context of the clonally integrated MCPyV genome in NE^hi^, *YAP1*-silenced MCC.

Several questions regarding YAP1- and WWTR1-mediated MCPyV T antigen repression and suppression of cell growth in NE^hi^ MCC remain. YAP1-TEAD and WWTR1-TEAD complexes have previously been reported to exhibit transcriptional repressor activity together with the NuRD complex (24, 77), and the related SV40 polyomavirus has a TEAD-binding motif within the NCCR of its genome (66, 67), leading us to wonder if MCPyV T antigen repression could be directly mediated by YAP1 or WWTR1. However, we did not observe a TEAD-binding sequence in the MCPyV NCCR, suggesting that repression may be an indirect effect. Furthermore, it is unclear why TEAD-binding–defective YAP1 and WWTR1 raise MCPyV LT levels. Depending on interacting partners, YAP1 and WWTR1 can have both transcriptionally repressive and activating functions (24, 25). It is possible that loss of TEAD binding could promote binding of YAP1 or WWTR1 to other transcriptionally activating partners that results in increased MCPyV LT levels either directly or indirectly. Further work will be needed to explore this idea. Finally, given the pleiotropic nature of the transcriptional changes induced in NE^hi^ MCCP cells by YAP1 and WWTR1 induction and the partial rescue of YAP1-dependent cell viability and S-phase population decreases, it will be important to investigate non–T antigen–dependent mechanisms of cell growth suppression in NE^hi^ MCC.

This study has provided insight into previously unrecognized subgroups of MCC and drawn clear connections to other NE cancers. We have provided a model by which classic and variant MCC cell lines can be classified according to NE marker and YAP1 and WWTR1 expression, and we suggest the importance of including variant MCC cell lines in future analyses. We have also provided molecular insight into the consequences of YAP1 and WWTR1 expression in NE^hi^ MCCP cells. Our findings in MCPyV-positive MCC cell lines tentatively support the previously proposed idea that reciprocal expression of YAP1 and WWTR1 and NE markers may be a pan-NE cancer phenomenon (40), but more work will be needed to confirm whether these effects also occur in NE^hi^ MCCN.

## Methods

### Tissue culture.

MKL-1, MKL-2, and MS-1 cell lines were a gift from Masahiro Shuda (University of Pittsburgh, Pittsburgh, Pennsylvania, USA). WaGa and UISO cell lines were a gift from Jürgen Becker (University Duisburg-Essen, Duisburg, Germany). PeTa and BroLi cell lines were a gift from Roland Houben (University of Würzburg, Würzburg, Germany). MCC13 and MCC26 cell lines were a gift from J. Helen Leonard (University of Western Australia, Crawley, Australia). HEK 293T cells were acquired from the American Type Culture Collection (ATCC). The MCC290, MCC301, MCC336, and MCC350 PDCLs were obtained in-house.

Established MCC cell lines were cultured in RPMI-1640 with glutamine (Gibco, Thermo Fisher Scientific) plus 10% FBS (MilliporeSigma) and 1% GlutaMAX (Thermo Fisher Scientific). 293T cells were cultured in DMEM with glutamine and sodium pyruvate (Gibco, Thermo Fisher Scientific) plus 10% FBS and 1% GlutaMAX. All cells tested negative for mycoplasma via PCR. 

PDCLs were cultured in non-TC–treated flasks in Neurocult NS-A medium plus 10% NS-A proliferation supplement (STEMCELL Technologies), 20 ng/mL human recombinant FGF (Thermo Fisher Scientific), 20 ng/mL human recombinant EGF (Thermo Fisher Scientific), 0.0002% heparin (STEMCELL Technologies), and 1% penicillin/streptomycin (Gibco, Thermo Fisher Scientific).

The isolation and derivation of several PDCLs used in this study, including verification as bona fide MCC cells, has been described elsewhere (44). This current work used identical conditions for de novo isolation of the following PDCLs: MCC410, MCC428, and MCC516. The PDCLs isolated in this study were confirmed as MCC by mRNA- and/or protein-level expression of common MCC markers including KRT20, the differential diagnostic marker for MCC. Briefly, to isolate single cells for scRNA-Seq and establishment of PDCLs, patient-derived tumor biopsies were minced into approximately 1 mm segments and incubated for several hours at 37°C in dissociation media (NS-A complete with 2 mg/mL each of collagenase IV and hyaluronidase; both from MilliporeSigma) with regular mixing. The tumor suspension was passed through a 100 μm filter to remove large debris and isolate single cells. For PDCL establishment, cells were plated and cultured as described above. For scRNA-Seq, cells were plated overnight and submitted for library preparation the next morning. For scRNA-Seq of MCC516, suspended and adherent cells were treated with Accutase (STEMCELL Technologies) and passed through a 70 μm filter to isolate single cells immediately prior to submission for library preparation.

### scRNA-Seq.

Sequencing libraries of single-cell preparations from tumor biopsies or MCC516s/a were generated with the 10x Genomics Chromium Next GEM Single Cell 5′ Kit, v2. cDNA libraries were sequenced on the Illumina NovaSeq S4 platform to an average depth of 20,000 reads per cell. MCC336 and MCC350 scRNA-Seq sequencing files were generated in a similar manner, as summarized in a separate publication (44). CellRanger was used to map reads to a hybrid of the Hg19 and MCPyV (R17b) genomes. Raw counts were input into Seurat, and high-quality cells were identified as having less than 10% mitochondrial reads, more than 500 total unique molecular identifiers (UMIs), and greater than 0.8 log_10_ genes per UMI. The resulting 38,077 cells from tumor biopsies and 9,150 cells from MCC516s/a cultures were analyzed in Seurat.

### Bulk tumor sample RNA-Seq.

Transcriptional analysis of the patient tumor biopsies has been described elsewhere (44). Briefly, patient tumor biopsies were either fresh-frozen and stored in liquid nitrogen or immediately treated with RNAlater (Thermo Fisher Scientific) and stored at –80°C. Each RNAlater-treated sample was homogenized with the QIAGEN TissueRuptor II, and RNA was isolated with the AllPrep DNA/RNA Mini Kit (QIAGEN). The NEBNext Ultra II RNA Library Prep Kit for Illumina was used to prepare sequencing libraries. Paired-end sequencing (PE150) was performed on the NovaSeq 6000 system (Illumina). Sequences were mapped and quantified to the decoy-aware, concatenated transcriptome of GRCh38.p13 (Ensembl, version 102) and MCPyV (R17b) using Salmon (78). Gene-level counts were generated via TxImport (79), and normalized counts were generated via DESeq2 (80). Samples were considered virus positive if the number of normalized MCPyV LT counts was greater than 100 and the number of normalized ST counts was greater than 10.

### Generation of plasmid constructs and YAP1- and WWTR1-expressing cell lines.

PLIX_402 (a gift from David Root, Addgene no. 41394) was used to generate inducible expression constructs. Constructs included YAP1 isoform 1-2 γ (from Addgene no. 124145, a gift from Megan Finch-Edmondson and Marius Sudol); YAP1 1-2 γ S94A, YAP1 1-2 γ 5SA (S61/109/127/164/397A); YAP1 1-2 γ 6SA (S61/94/109/127/164/397A); WWTR1 (from Addgene no. 82253, a gift from Jesse Boehm, William Hahn, and David Root); WWTR1 F52A, WWTR1 4SA (S66/89/117/311); WWTR1 4SA/F52A; or EGFP. pLenti CMV Blast DEST (a gift from Eric Campeau and Paul Kaufman, Addgene no. 17451) was used to generate constructs, which contained MCPyV L21–truncated LT (NCBI database accession no. KC426955) or MCPyV L21–truncated LT E216K. 293T cells were transfected using polyethyleneimine with either pLIX_402 or pLenti constructs in addition to psPAX2 and pMD2.G (gifts from Didier Trono, Addgene nos. 12260 and 12259). Cells were transduced with lentivirus-containing supernatants by spinfection at 931*g* for 1 hour (WaGa) or 2 hours (MKL-1) at room temperature with 2 μg/mL polybrene (MilliporeSigma). After spinfection, 1 volume of complete RPMI was added. Cells were selected after 24 hours with 1 μg/mL puromycin (Thermo Fisher Scientific) for 3 days (WaGa) or 4 days (MKL-1) or with 20 μg/mL blasticidin (Thermo Fisher Scientific) for 6 days.

### MKL-1 YAP1 and WWTR1 RNA-Seq.

YAP1- or WWTR1-expressing MKL-1 cells were treated with 2 μg/mL DOX (MilliporeSigma) for 3 days, and RNA was extracted with TRIzol plus chloroform (Thermo Fisher Scientific). Library preparation and sequencing were performed by Novogene. Samples were sequenced on an Illumina NovaSeq 6000 to obtain paired-end 150 bp sequences. Sequences were mapped and quantified as described above. Differential expression analysis was performed via DESeq2 with comparisons of each condition with GFP. To obtain corrected target genes, lists of up- and downregulated DEGs versus GFP were compared between YAP1 and YAP1 S94A or WWTR1 and WWTR1 F52A, and overlaps were removed from the YAP1 and WWTR1 lists. 

### Viability assays.

YAP1- or WWTR1-expressing MKL-1 cells were treated with 2 μg/mL DOX for 3 and 6 days with complete refreshment on day 3. At each time point, cells were treated with Accutase to disrupt clumps, and suspensions were either plated in 96-well plates for a CellTiter-Glo viability assay (Promega) or lysed for SDS-PAGE.

### Cell-cycle analysis.

MKL-1 cells with inducible YAP1 or WWTR1 were treated with 2 μg/mL DOX for 6 days with refreshment on day 3. On the final day, cells were labeled with 10 μM 5-ethynyl-2′-deoxyuridine (EdU) (Click Chemistry Tools) in a final concentration of 0.1% DMSO for 1 hour prior to harvesting. Cells were dissociated into single cells with Accutase, and 2 million cells per sample were fixed in in sub-zero (–20°C) 70% ethanol. The remaining cells were lysed. Alexa Fluor 647 azide (Thermo Fisher Scientific) was conjugated onto incorporated EdU via copper I–catalyzed click chemistry. Cells were stained with DAPI (MilliporeSigma) to measure total DNA content. Stained cells were passed through a 70 μm filter, and flow cytometry was performed for cell-cycle analysis. Doublets were removed via DAPI height versus area discrimination. A minimum of 30,000 events were recorded per sample.

### IHC.

IHC was performed on the Leica Bond automated staining platform using the Leica Biosystems Refine Detection Kit. Antibodies and dilutions used for IHC include: 1:200 rabbit anti-ATOH1 (Proteintech, 21215-1-AP) and 1:100 mouse anti-YAP1 (Santa Cruz Biotechnology, SC101199), each with citrate antigen retrieval; and 1:100 rabbit anti-TAZ (Cell Signaling Technology, 72804) and 1:50 mouse anti-KRT20 (DAKO, 72804), each with EDTA antigen retrieval.

### Immunoblotting.

Cell pellets were lysed in RIPA buffer plus protease and phosphatase inhibitors. Lysates were run on a gradient SDS-PAGE gel, followed by transfer onto a PVDF membrane and blocking in 5% milk. Blots were incubated at 4°C overnight in primary antibody and imaged with SuperSignal West Femto (Thermo Fisher Scientific) substrate (full, uncut gels are available in the Supplemental Material). Primary antibodies and concentrations used in this study include: 1:1,000 rabbit anti-YAP (Cell Signaling Technology, 14074); 1:1,500 rabbit anti-YAP/TAZ (Cell Signaling Technology, 8418); 1:1,000 rabbit anti-LATS1 (Cell Signaling Technology, 3477); 1:1,000 rabbit anti-LATS2 (Cell Signaling Technology, 5888); 1:1,000 rabbit anti-MST1 (Cell Signaling Technology, 3682); 1:1,000 rabbit anti-MST2 (Cell Signaling Technology, 3952); 1:1,000 rabbit anti–pT35 MOB1 (Cell Signaling Technology, 8699); 1:1,000 rabbit anti-MOB1 (Cell Signaling Technology, 13730); 1:1,500 rabbit anti-TBP (Cell Signaling Technology, 8515); 1:30,000 mouse anti-vinculin (MilliporeSigma, V9131); 1:1,000 rabbit anti-ATOH1 (Proteintech, 21215-1-AP); 1:500 mouse anti-SOX2 (R&D Systems, MAB2018); 1:1,000 rabbit anti-CHGA (Cell Signaling Technology, 60893); 1:1,000 rabbit anti-SYP (Cell Signaling Technology, 5461); 1:500 mouse anti-KRT20 (Thermo Fisher Scientific, MS-377-S0); 1:500 mouse Ab5 (64, 68); and 1:1,000 rabbit anti-GFP (Cell Signaling Technology, 2956).

### RT-qPCR.

To determine baseline *YAP1* and *WWTR1* expression in cell lines, RNA was extracted with the QIAGEN RNeasy kit. To measure expression changes in YAP1- and WWTR1-expressing MKL-1 cells, cells were treated with 2 μg/mL DOX for 3 days, and RNA was extracted with TRIzol plus chloroform. Reverse transcription was carried out with the Applied Biosystems High-Capacity cDNA Reverse Transcription Kit. RT-qPCR was performed using Brilliant III Ultra-Fast Sybr Green QPCR Master Mix (Agilent Technologies) with 40 cycles of 2-step amplification at 60°C. Data were analyzed using the ΔΔCt method with normalization to 18S rRNA. The primers used in this study include: 18S rRNA (5′-AACCCGTTGAACCCCATT-3′, 5′-CCATCCAATCGGTAGTAGCG-3′), MCPyV LT (5′-ATTCAGCTTCGGGAAGGCATAC-3′, 5′-GCTCCCCTGGATGCATTGG-3′), and MCPyV ST (5′-AGGTCCTGGGTGCATGCTT-3′, 5′-ACACTCTCCCCACGTCAGAC-3′).

### Microscopy.

Bright-field microscopy images were taken at ×4 or ×10 magnification using SPOT5.2 software on a Nikon Eclipse TE300 camera inverted microscope with a Diagnostic Instruments Model no. 25.4 2 Mp Slider Camera.

### Data availability.

MKL-1 YAP1 and WWTR1 transcriptional profiling data are available through NCBI’s Gene Expression Omnibus (GEO) database (accession no. GSE189054). The bulk tumor biopsy and PDCL RNA-Seq data sets are described in a separate publication (44). They and the scRNA-Seq data sets are available through the NCBI’s Database of Genotypes and Phenotypes (dbGaP) (accession no. phs002260).

### Statistics.

All statistical tests were performed using GraphPad Prism (GraphPad Software). A *P* value of less than 0.05 was considered statistically significant. The statistical tests used to determine significance include 2-way, repeated-measures ANOVA with Tukey’s post hoc test, 1-way ANOVA with Dunnett’s post hoc test, and the log-rank test. The demographics table and swimmer plots were generated using R (version 4.2.1). Survival analysis and KM plots were generated using SAS, version 9.4.

### Study approval.

This study was approved by the IRB of DFCI for the use of human tumor samples (IRB protocol no. 09-156). Written informed consent was received prior to patient participation in the study.

## Author contributions

TCF and AKG designed research studies, conducted experiments, analyzed data, and wrote the manuscript. ML and AGH performed analyses of the clinical metadata. JC conducted experiments. HD and MT collected tumor samples and provided patient metadata. DBK and CJW provided PDCLs and primary cell bulk RNA-Seq data. JAD conceptualized and supervised the study. Co–first authorship order was decided on the basis of TCF’s completion of the revisions.

## Supplementary Material

Supplemental data

Trial reporting checklists

ICMJE disclosure forms

Supplemental table 1

## Figures and Tables

**Table 1:**
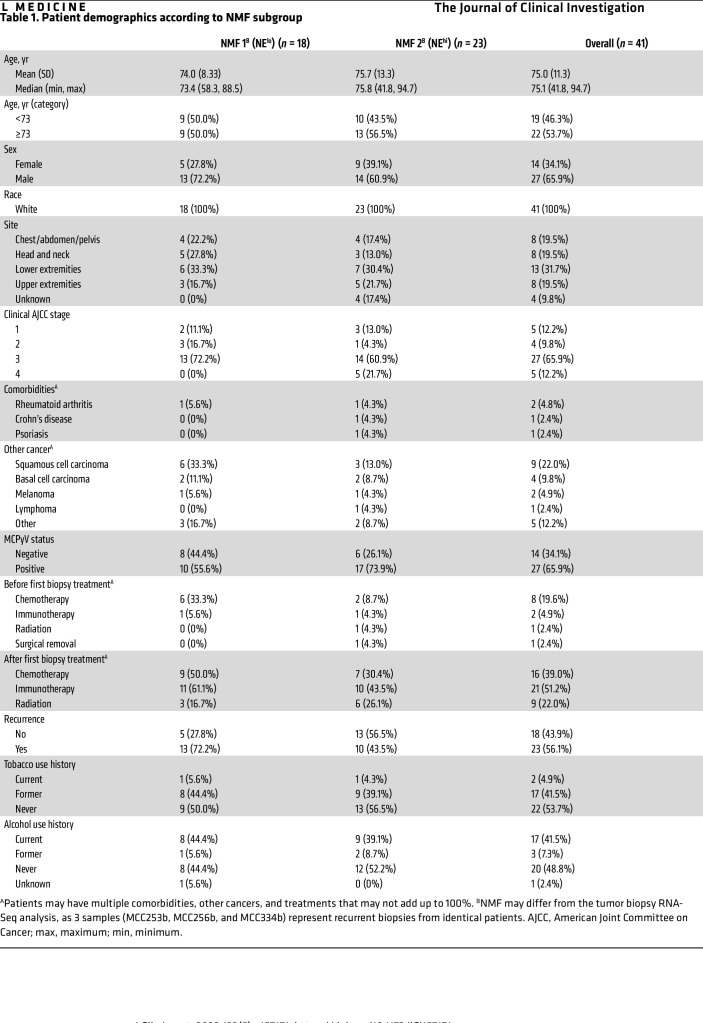
Patient demographics according to NMF subgroup

## Version 1 01/31/2023

In-Press Preview

## Version 2 03/01/2023

Electronic publication
