# Supplementary material for: YAP1 and WWTR1 expression inversely correlates with neuroendocrine markers in Merkel cell carcinoma
Source: J Clin Invest. 2023 Mar 1;133(5):e157171. doi: 10.1172/JCI157171 (PMC9974098; doi:10.1172/JCI157171)

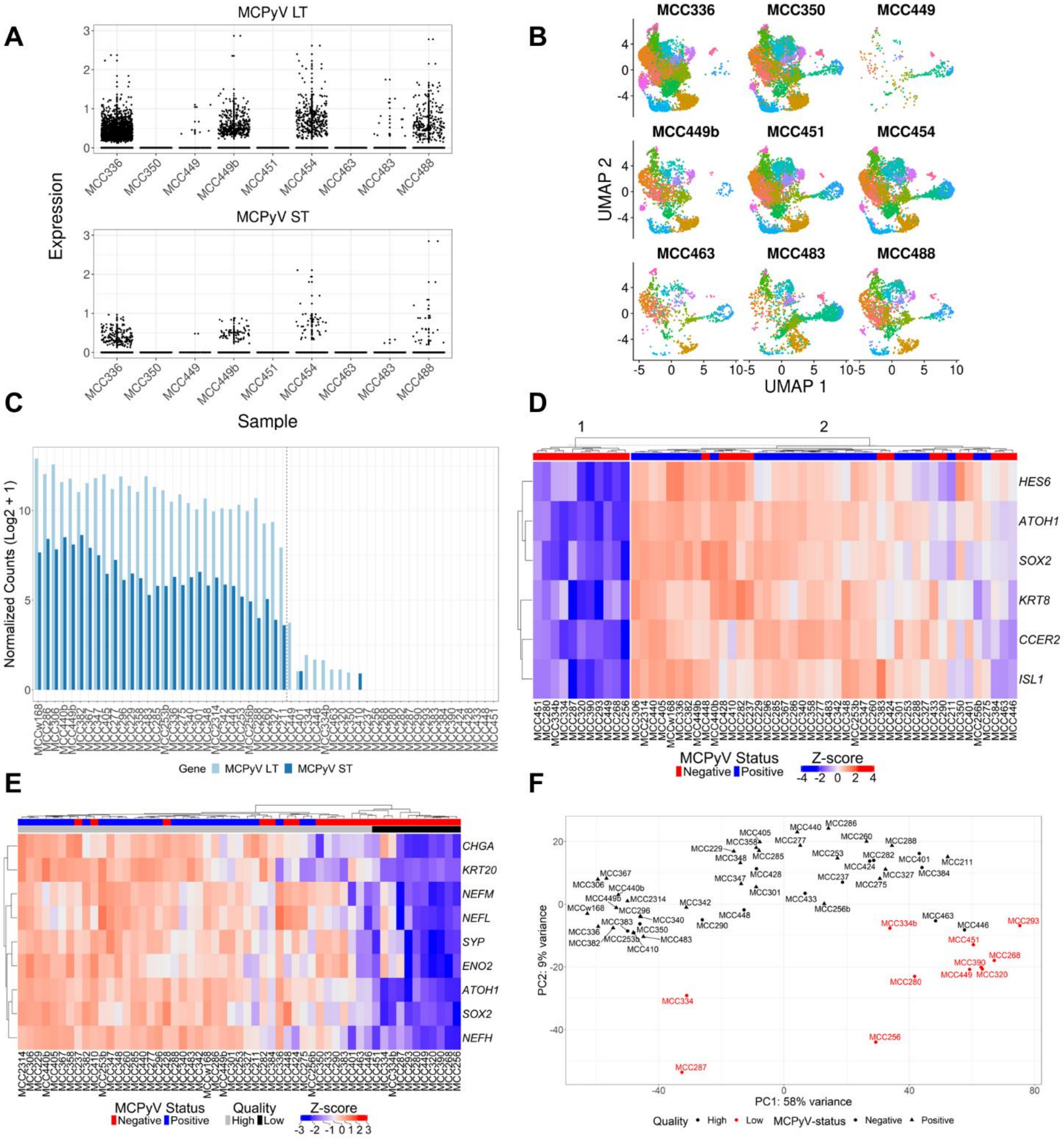

Figure S1. Viral-status determination and quality control for scRNA-seq and bulk tumor biopsy RNA-seq. A. MCPyV *LT* and *ST* normalized expression in each tumor biopsy assayed by scRNA-seq. Samples with nonzero expression of both *LT* and *ST* were deemed virus-positive. B. DimPlot of cells representing clusters present within each of 9 tumor samples assayed by scRNA-seq. C. Log2 + 1 normalized counts of MCPyV *LT* and *ST* transcripts in each of the 55 tumor samples analyzed by bulk RNA-seq. Cutoff of 100 normalized *LT* counts and 10 normalized *ST* counts were used to call tumors virus-positive or virus-negative. Line denotes samples meeting the cutoff. D. Heatmaps for expression of cluster 0 and E. other NE/MCC genes in the 55 bulk RNA-sequenced tumor samples. The 11 samples which lacked robust expression of the cluster 0 and canonical MCC genes (black) were removed from further analysis. F. PCA of all 55 bulk RNA-sequenced tumor samples highlighting the 11 samples removed (red).

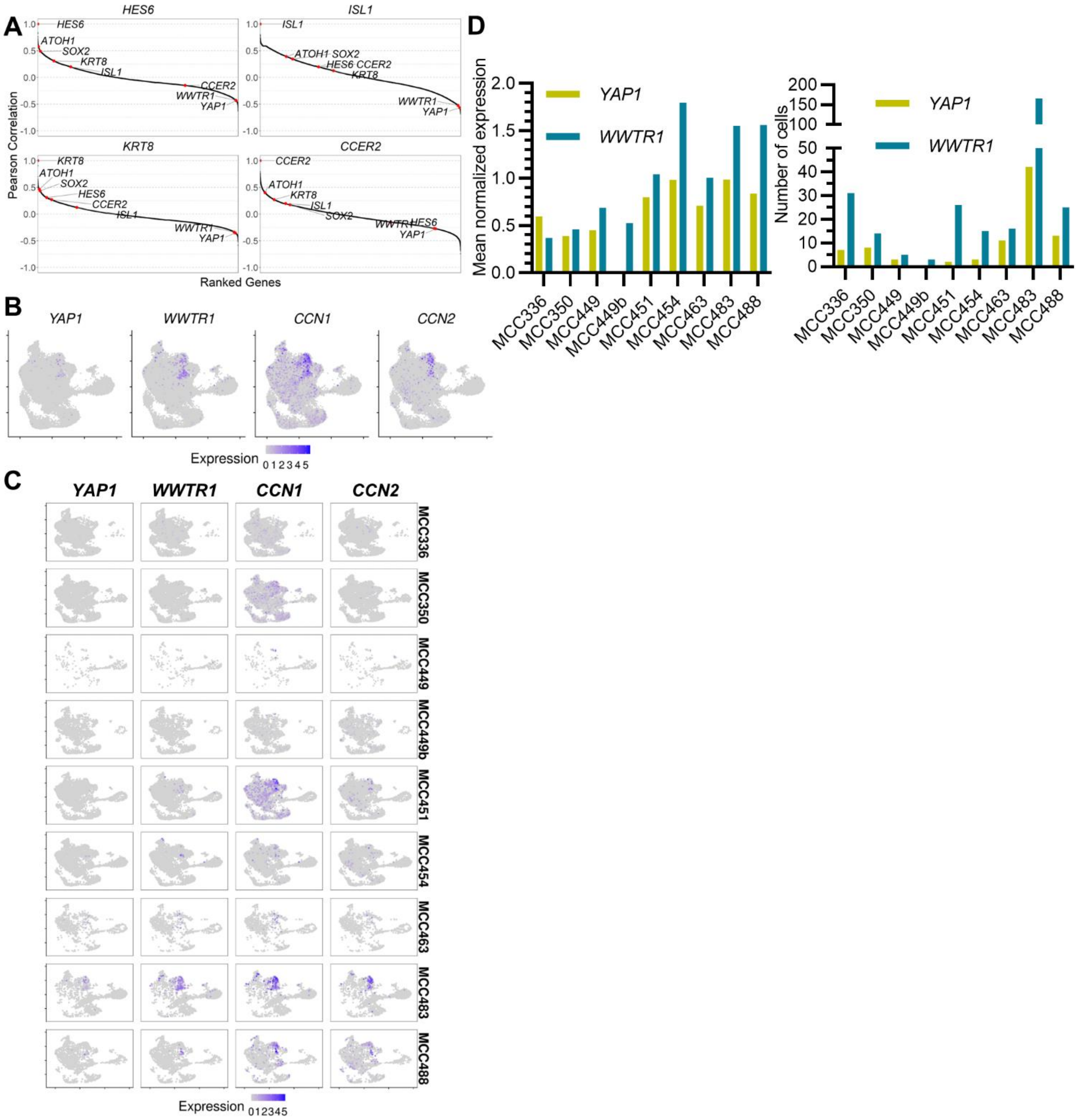

Figure S2. Analyses of *YAP1/WWTR1* expression in patient-derived MCC tumor biopsies. A. Genome-wide gene-to-gene Pearson correlations for 44 bulk RNA-sequenced tumor samples highlighting correlations of *HES6, ISL1, KRT8,* and *CCER2* with *YAP1* and *WWTR1* across all samples. B. Feature plots showing expression of *YAP1, WWTR1,* and their targets *CCN1* and *CCN2* in the scRNA-seq dataset. C. Feature plots of *YAP1, WWTR1, CCN1,* and *CCN2* in each of the 9 tumor samples assessed by scRNA-seq. D. Number of *YAP1*- and *WWTR1*-expressing cells and mean normalized expression values for positive cells across all samples.

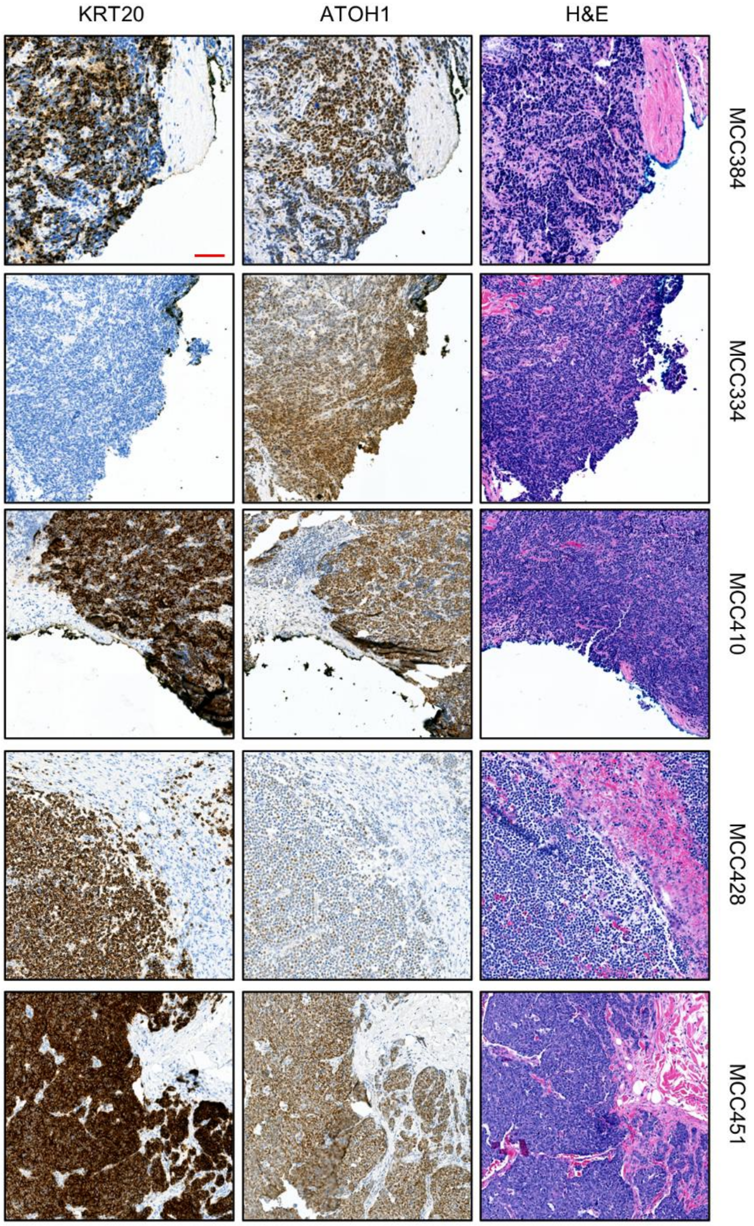

Figure S3. Immunohistochemistry of patient-derived tumor biopsies for hematoxylin and eosin, ATOH1, and KRT20 markers. Images were captured at 20x magnification. Red scale bar represents 100 µm.

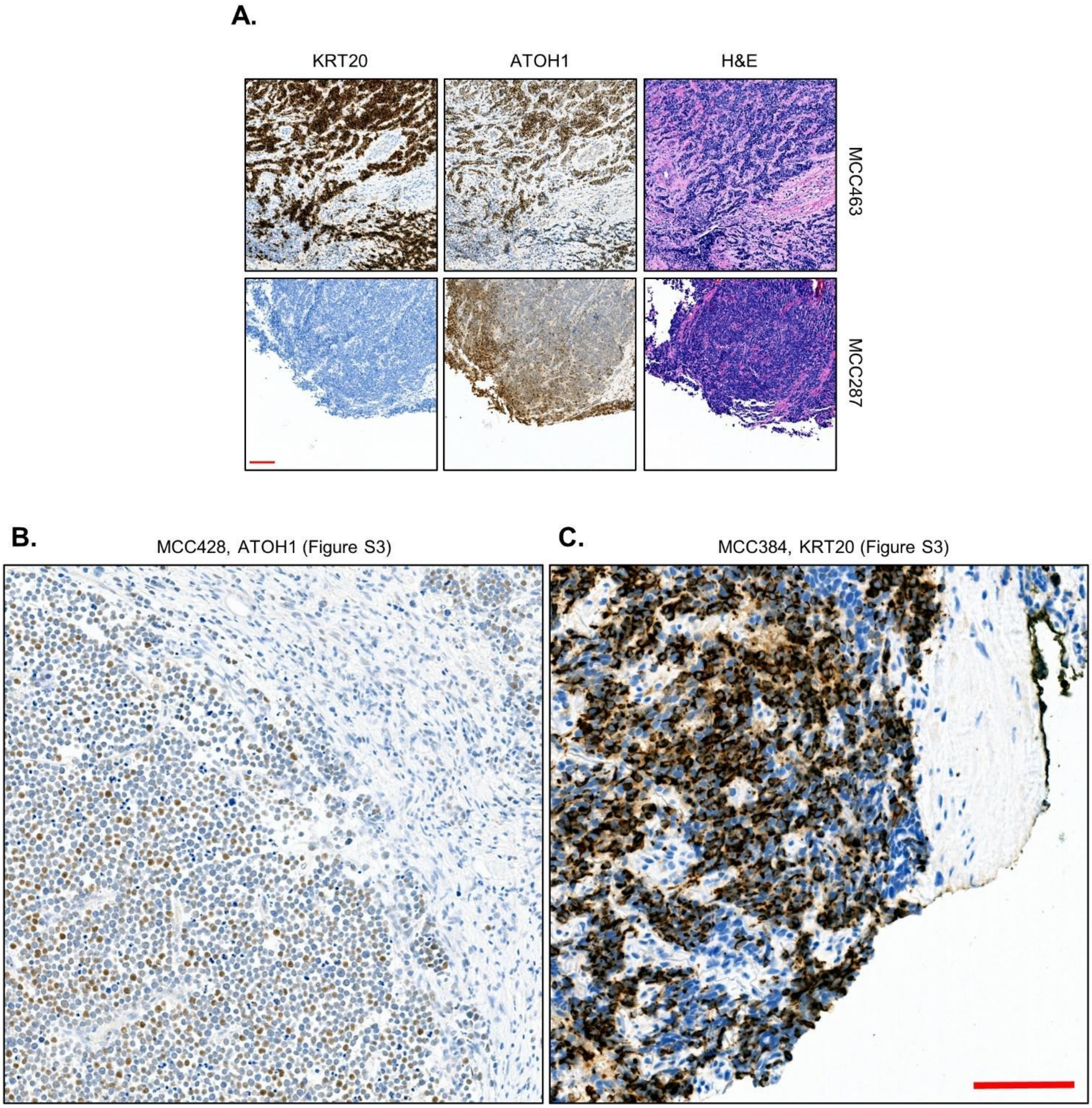

Figure S4. Additional immunohistochemistry analysis of patient-derived tumor biopsies. A. Images processed and analyzed as in Figure S3. B. Zoom and blowup of representative tumor section in MCC428 for ATOH1 staining. 20x magnification; identical to Figure S3. C Zoom and blowup of representative tumor section of MCC384 for KRT20 staining. 20x magnification; identical to Figure S3. Red scale bar represents 100 µm.

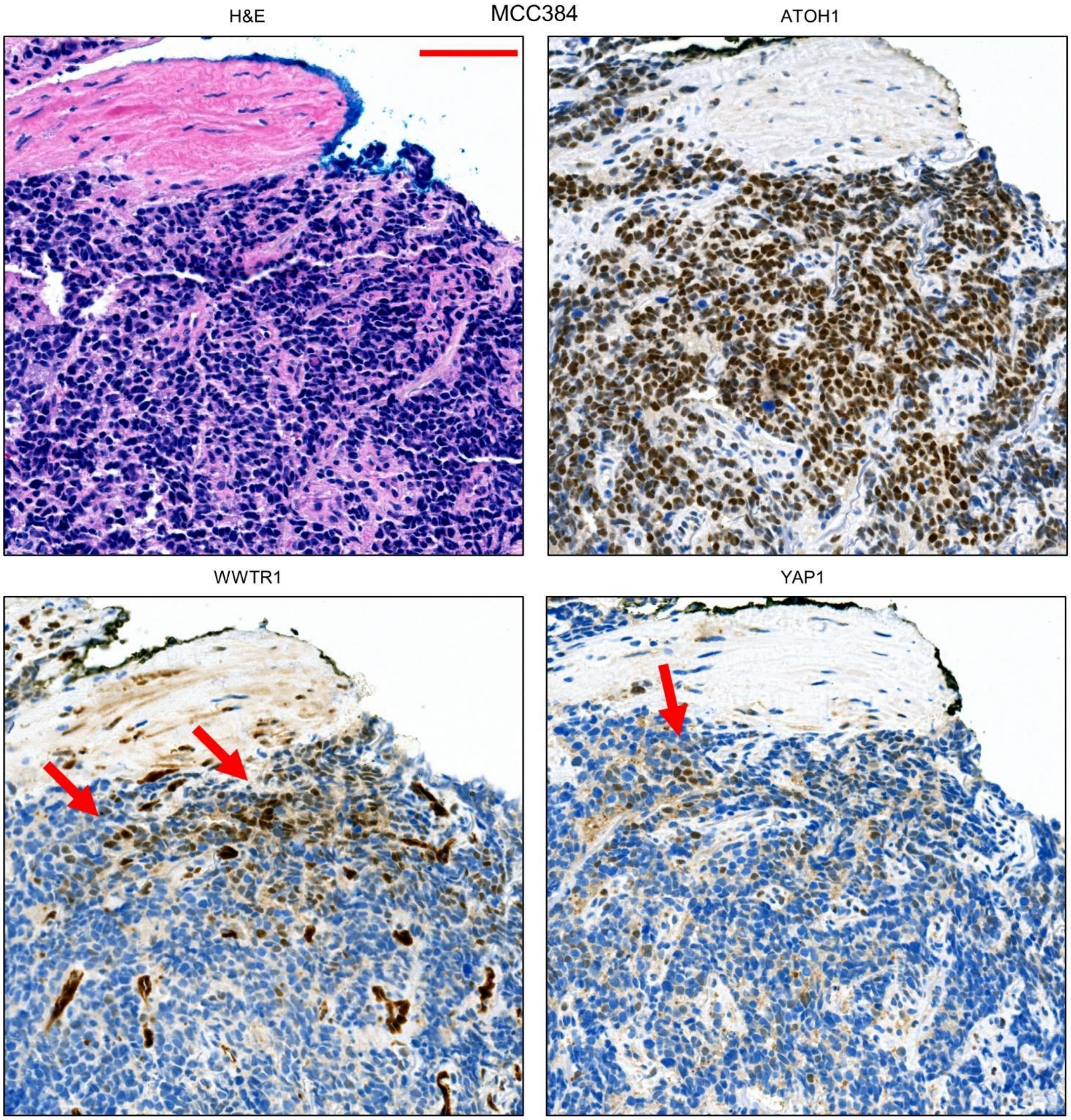

Figure S5. Immunohistochemistry of WWTR1- and YAP1-positive tumor biopsy section. Images are from MCC384 including H&E (top left), ATOH1 (top right), WWTR1 (bottom left), and YAP1 (bottom right). Magnification 20x. Red scale bar represents 100 µm. Red arrows denote WWTR1- or YAP1-positive cells. The H&E and ATOH1 stain images are identical to Figure S3, top row.

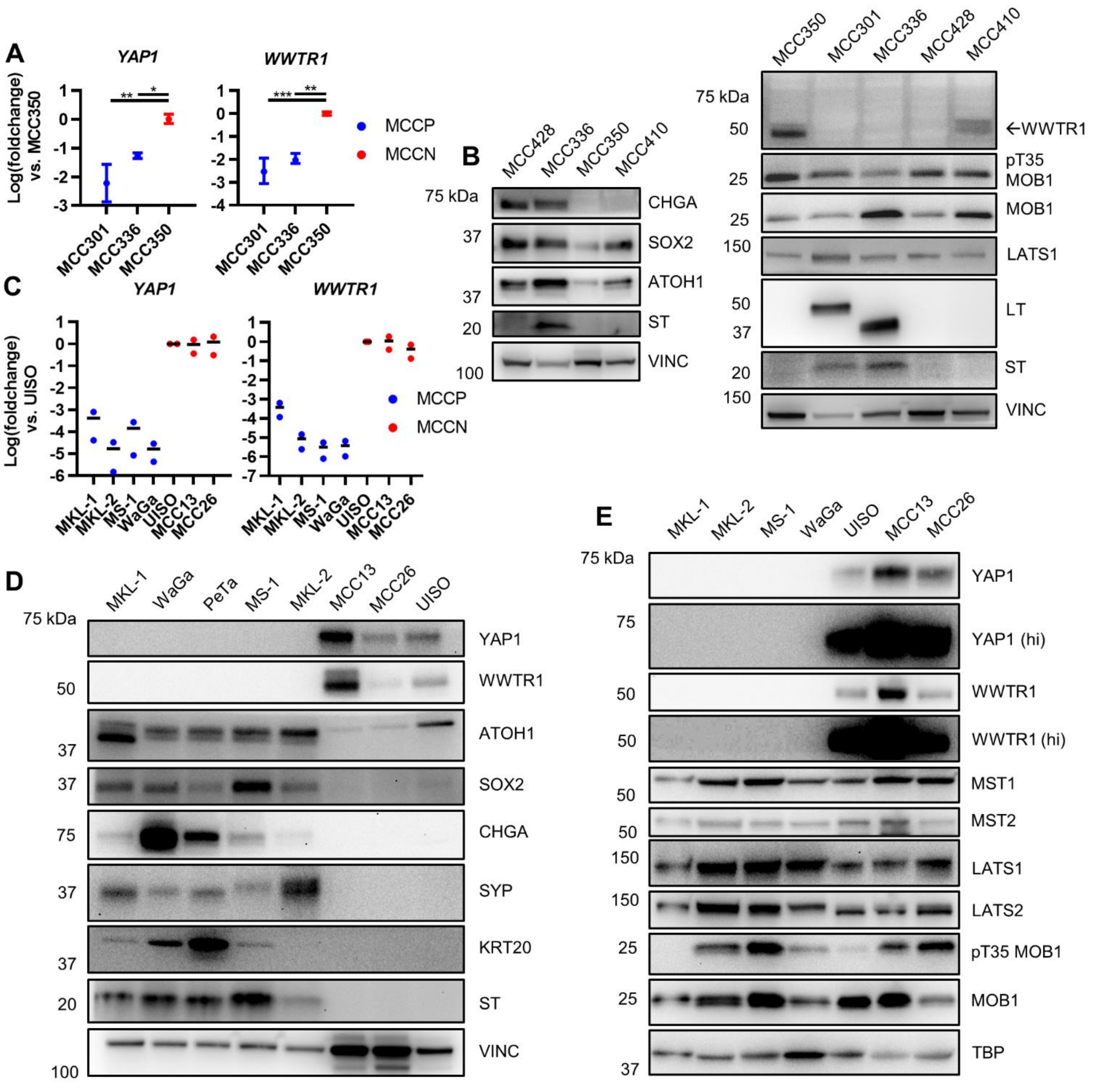

Figure S6. Analyses of *YAP1/WWTR1* expression in PDCLs and established MCC cell lines. A. RT-qPCR of *YAP1/WWTR1* in PDCLs. N = 3; mean + CI from technical replicate SD; normalized to 18S rRNA then MCC350; one-way ANOVA with Dunnett post-hoc tests on delta CT values; * $p < 0.05$, ** $p < 0.01$, *** $p < 0.001$. B. Immunoblots of WWTR1, NE markers, Hippo pathway kinase LATS1, and MOB1 in PDCLs. N = 1. C. RT-qPCR of *YAP1/WWTR1* in established MCC cell lines. N = 2; mean shown; normalized to 18S rRNA then UISO. D. Immunoblot of established MCC cell lines for YAP1/WWTR1 and NE markers. Representative of 3 images. E. Immunoblot of the Hippo pathway kinases and MOB1 in established MCC cell lines. Representative of 2 images.

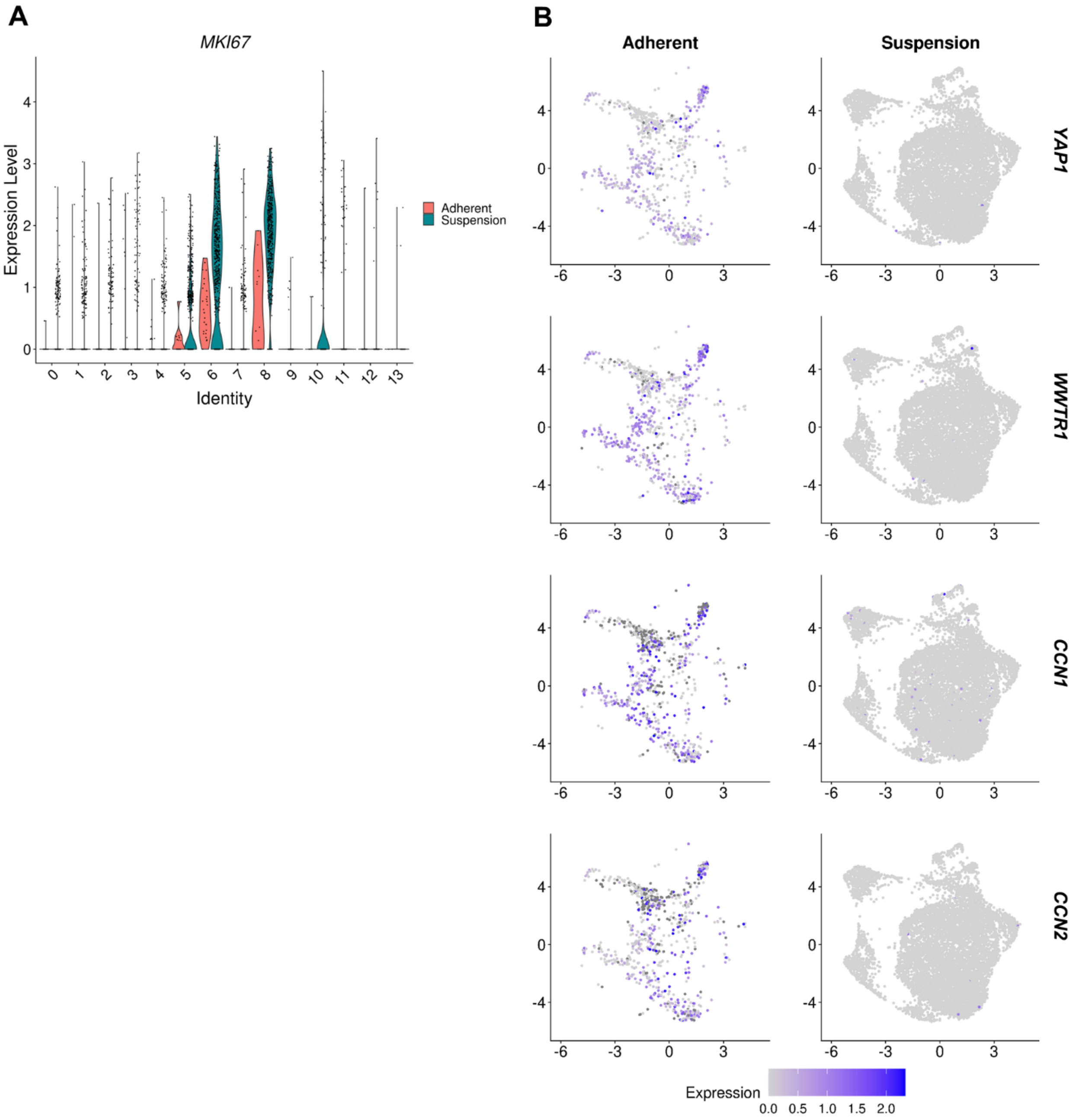

Figure S7. Additional scRNA-seq characterization of MCC516s/a. A. Violin plots of normalized *MKI67* expression in each cluster for MCC516s/a. B. Feature plots of normalized expression of *YAP1, WWTR1, CCN1,* and *CCN2* in MCC516s/a.

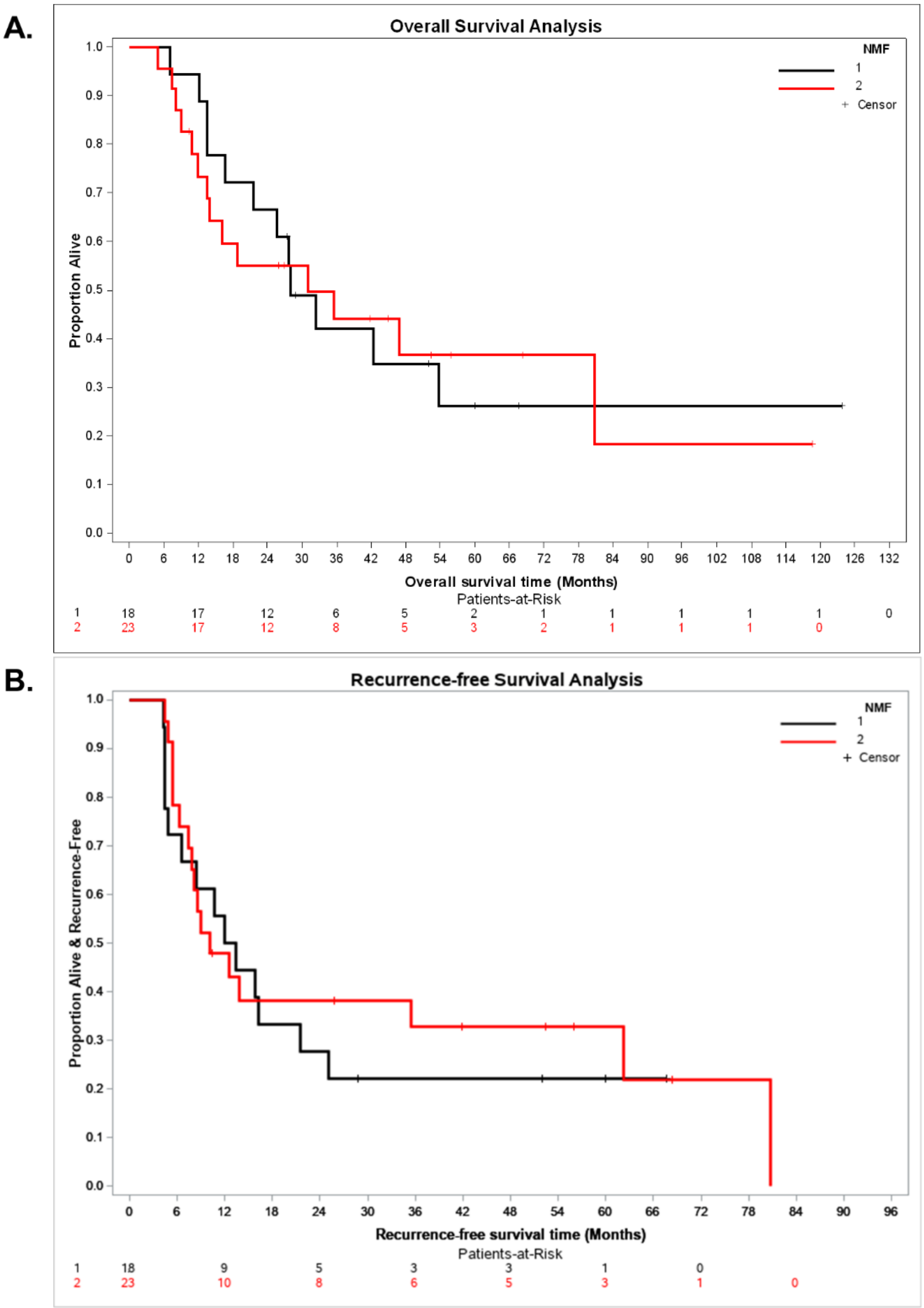

Figure S8. Patients with tumors in NMF groups 1 and 2 did not significantly differ in overall or recurrence-free survival. A. Kaplan-Meier (KM) overall survival (OS) curves of patient metadata stratified on NMF subgroups identified in the bulk tumor biopsy RNA-seq dataset. OS was measured from time of patient inclusion in the study until death or study termination. The median OS was 28.1 and 31 months for NMF group 1 and 2, respectively. At 60 months, the OS (95% CI) was 26% (11-63%) and 37% (20-67%) for NMF group 1 and 2 respectively. CI was calculated with log transformation. NMF group 1 and 2 did not differ significantly in terms of OS (P-value = 0.76, Logrank test, stratified by age (>= 73 vs. <73)). B. KM recurrence-free survival (RFS) curves of patient metadata stratified on NMF subgroups identified in the bulk tumor biopsy RNA-seq dataset. RFS was measured from time of patient inclusion in the study until the first noted recurrence or study termination. The median RFS was 12.7 and 10.1 months for NMF group 1 and 2, respectively. At 60 months,

the RFS (95% CI) was 22% (9-53%) and 33% (18-60%) for NMF group 1 and 2, respectively. NMF group 1 and 2 did not differ significantly in terms of RFS (*P*-value 0.70; Logrank test, stratified by age (>= 73 vs. <73)).

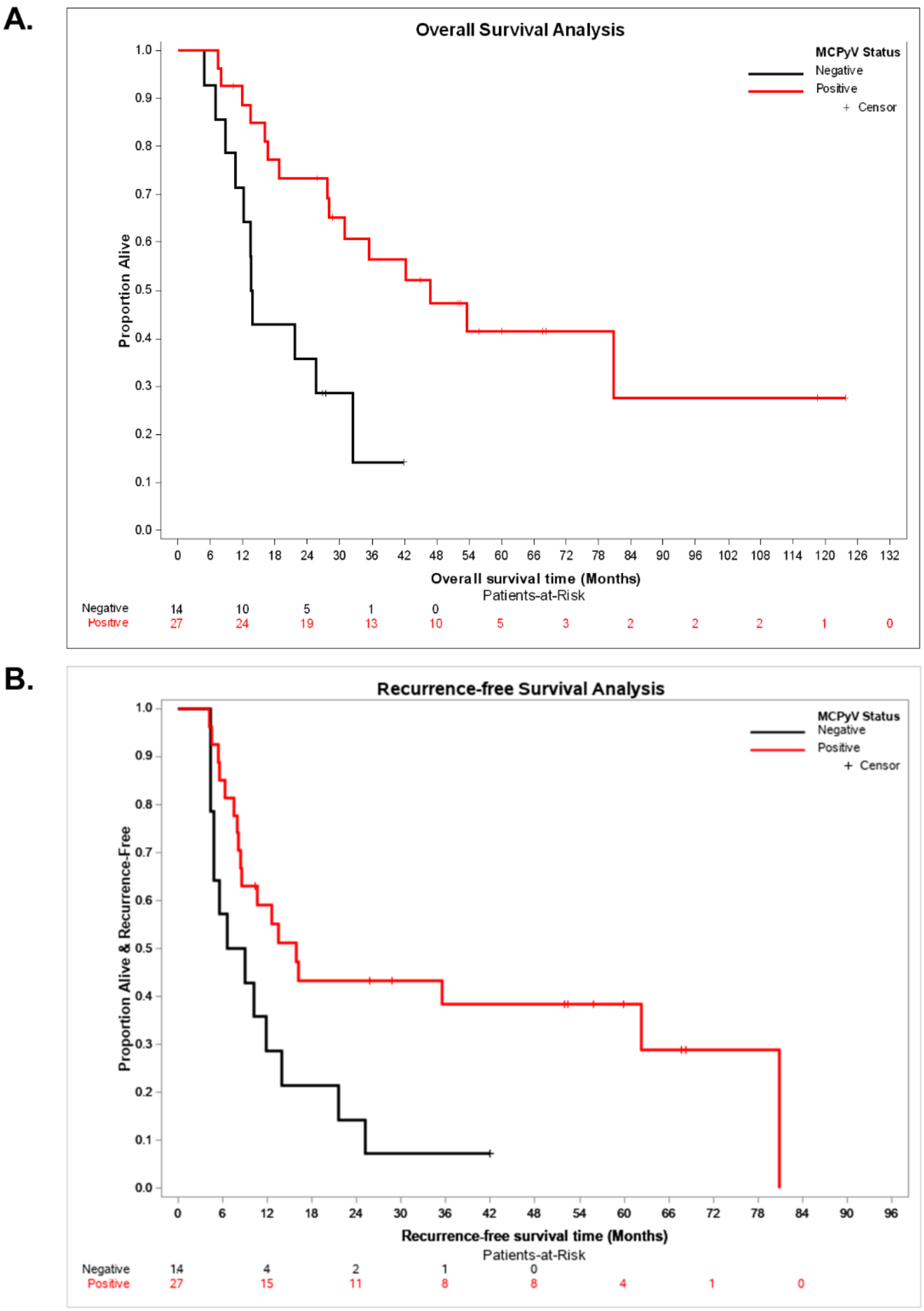

Figure S9. MCPyV-positive patients had significantly increased overall survival and recurrence-free survival as compared to MCPyV-negative patients. A. KM OS curves of patient metadata stratified on MCPyV-status. OS was measured from time of patient inclusion in the study until death or study termination. The median OS was 13.7 and 46.8 months for MCPyV-negative and -positive groups, respectively. At 60 months, the OS (95% CI) was not evaluable and 41% (25-68%) for MCPyV-negative and -positive patients, respectively. MCPyV-positive patients had significantly increased OS as compared to MCPyV-negative *(P*-value < 0.01; Logrank test, stratified by age (>= 73 vs. <73)). B. KM RFS curves of patient metadata stratified on MCPyV-status. RFS was measured from time of patient inclusion in the study until the first noted recurrence or study termination. The median RFS was 7.8 and 15.8 months for MCPyV-negative and -positive samples, respectively. At 60 months, the RFS (95% CI) was not evaluable and 38% (23-63%) for MCPyV-negative and -positive patients, respectively. MCPyV-positive patients had significantly increased RFS as compared to MCPyV-negative (*P*-value < 0.05; Logrank test, stratified by age (>= 73 vs. <73)).

A.

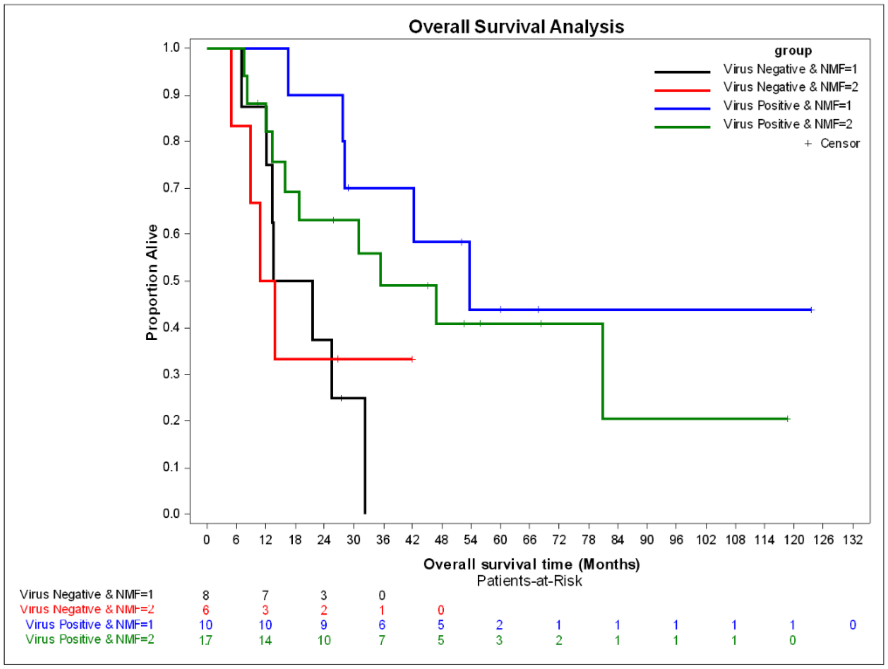

B.

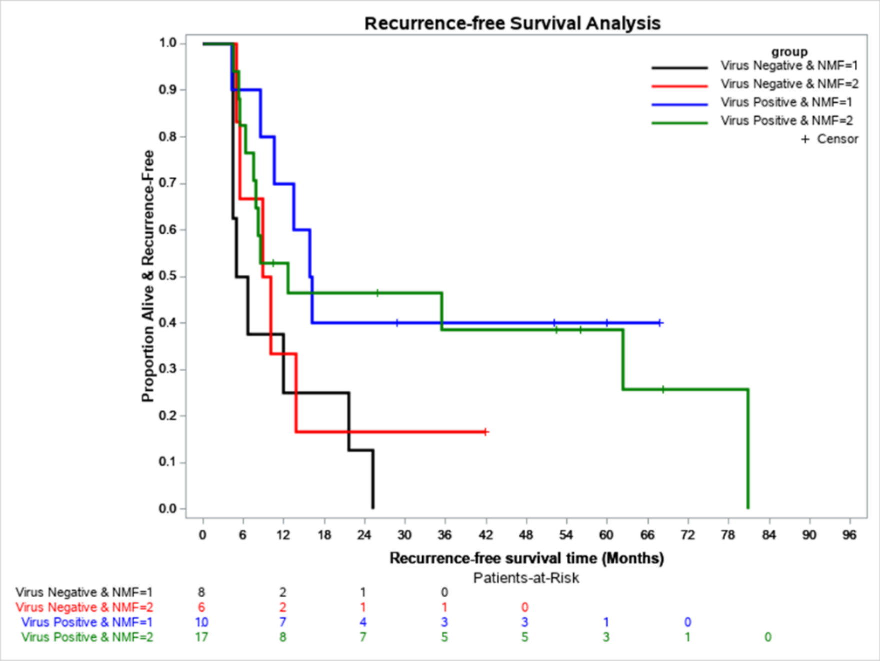

Figure S10. NMF-status and viral-status did not significantly interact in overall survival and recurrence-free survival analyses of patient metadata. A. KM OS curves of patient metadata stratified on NMF-group and MCPyV-status. OS was measured from time of patient inclusion in the study until death or study termination. The median OS (months) was 17.6 for NMF1/MCCN, 12.3 for NMF2/MCCN, 53.6 for NMF1/MCCP, and 35.5 for NMF2/MCCP. At 60 months, the OS (95% CI) was not evaluable for NMF1/MCCN and NMF2/MCCN, 44% for NMF1/MCCP (20-96%), and 41% for NMF2/MCCP (22-76%). There was no significant interaction between NMF- and MCPyV-subgroups in terms of OS (*P*-value = 0.07, Logrank test, stratified by age (>= 73 vs. <73)). B. KM RFS curves of patient metadata stratified on NMF-group and MCPyV-status. RFS was measured from time of patient inclusion in the study until the first noted recurrence or study termination. The median RFS (months) was 5.7 for NMF1/MCCN, 9.5 for NMF2/MCCN, 16.0 for NMF1/MCCP, and 12.5 for NMF2/MCCP. At 60 months, the RFS (95% CI) was not evaluable for NMF1/MCCN and NMF2/MCCN, 40% for NMF1/MCCP (19-85%) and 39% for NMF2/MCCP (21-73%). There was no significant

interaction between NMF- and MCPyV-subgroups in terms of RFS (*P*-value = 0.13, Logrank test, stratified by age (>= 73 vs. <73)).

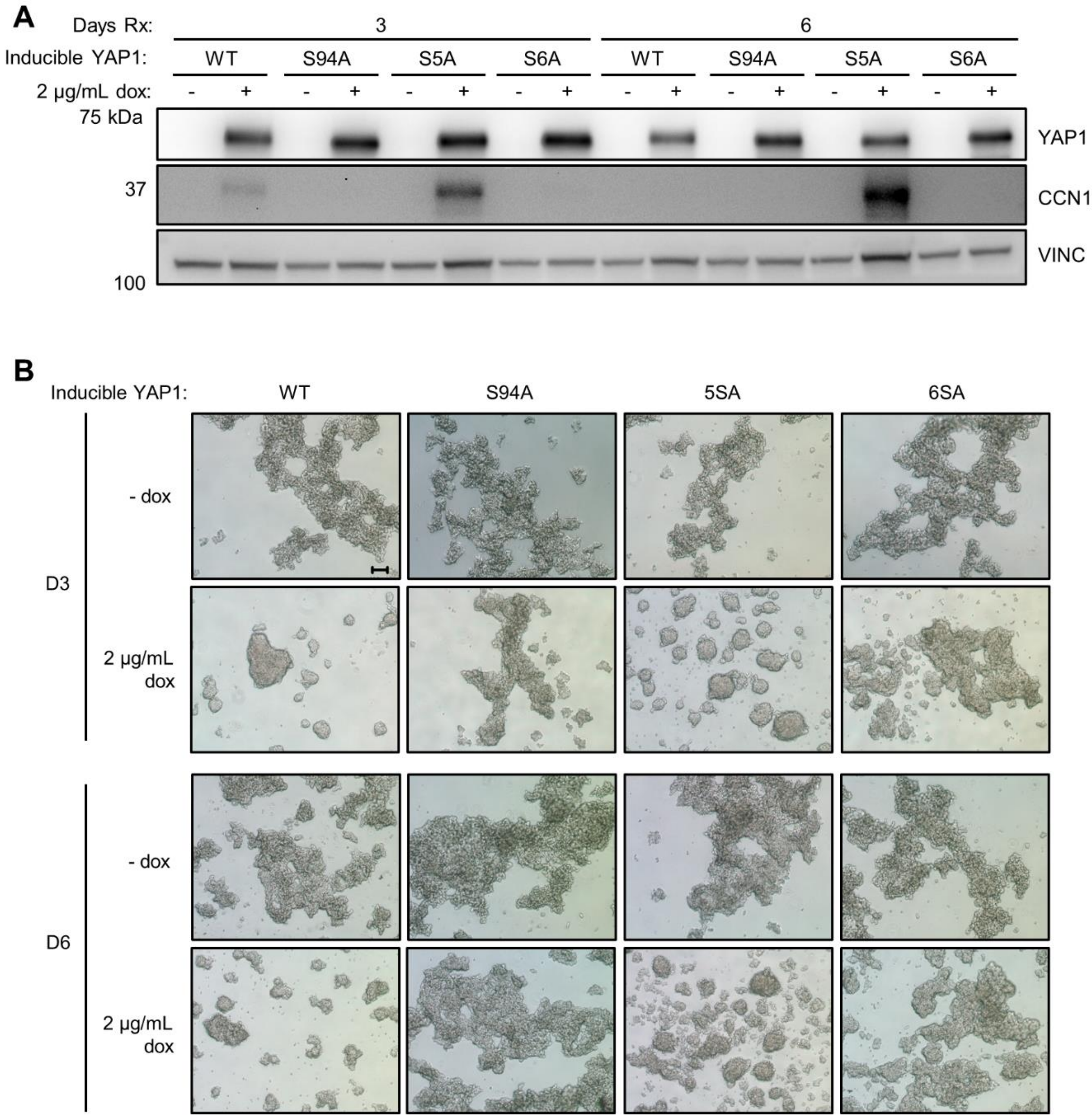

Figure S11. Transcriptionally active YAP1 expression in NE-hi MCCP cells induces CCN1 expression and adhesion-related morphological changes. A. Immunoblots of MKL-1 cells induced to express YAP1 for 3 or 6 days. Representative of 3 images. B. Bright-field image of MKL-1 cells upon expression of wild-type or mutant YAP1 for 3 or 6 days. 4x magnification; scale bar 100 µm. Representative of 3 experiments.

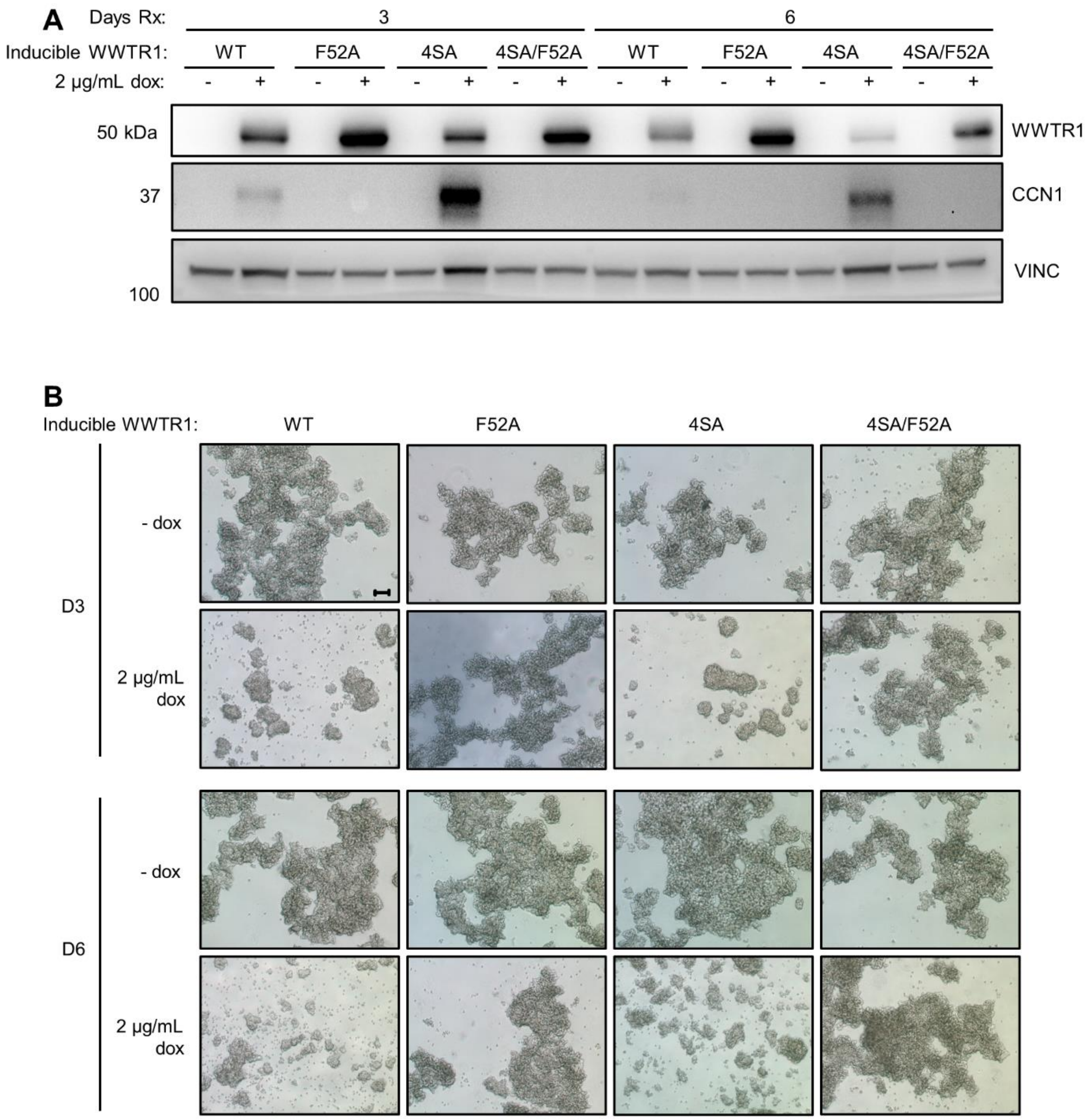

Figure S12. Transcriptionally active WWTR1 expression in NE-hi MCCP cells induces CCN1 expression and adhesion-related morphological changes. A. Immunoblots of MKL-1 cells induced to express WWTR1 for 3 or 6 days. Representative of 3 images. B. Bright-field image of MKL-1 cells upon expression of wild-type or mutant WWTR1 for 3 or 6 days. 4x magnification; scale bar 100 µm. Representative of 3 experiments.

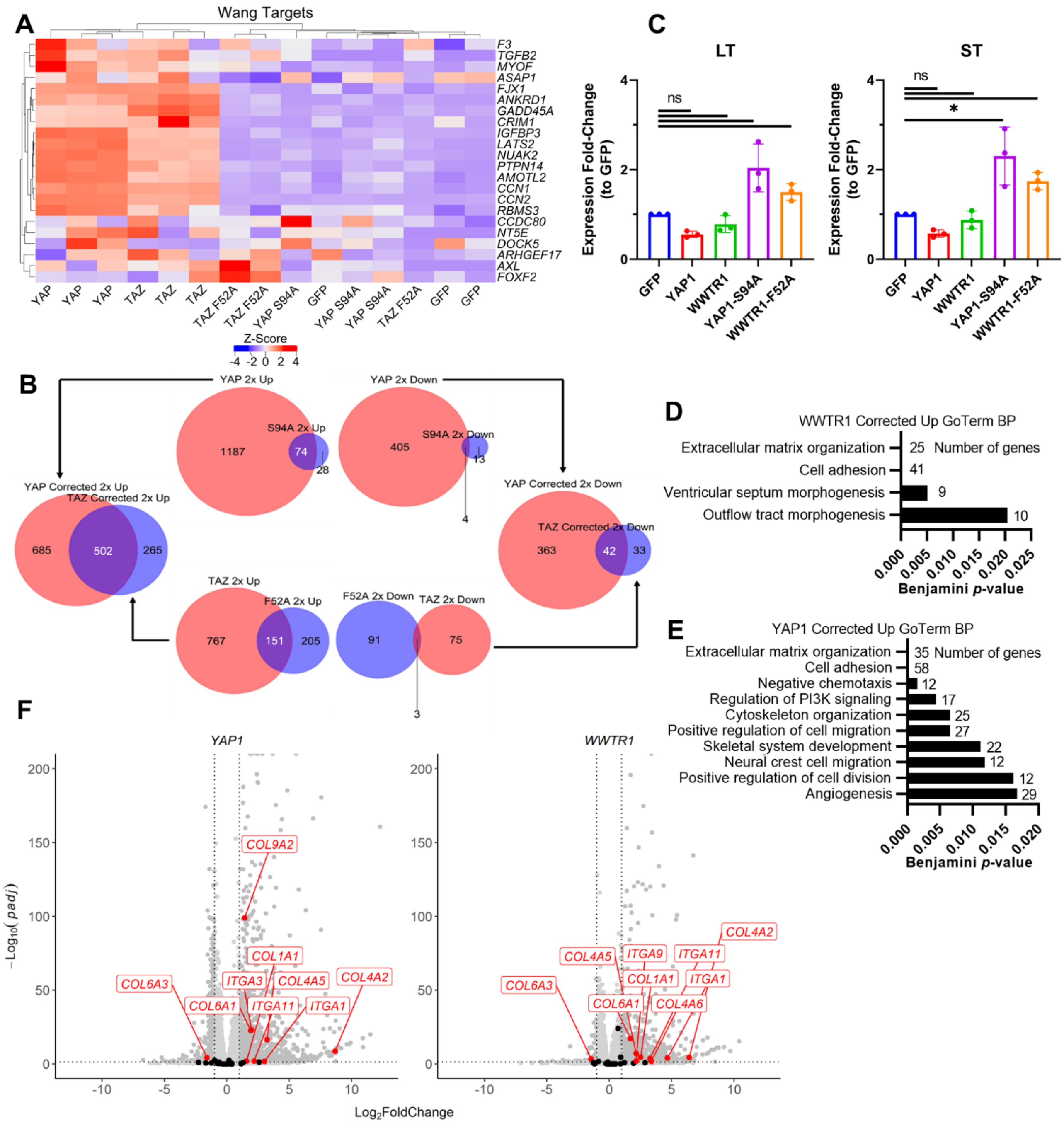

Figure S13. Quality control and correction strategy for RNA-seq of MKL-1 cells expressing YAP1 or WWTR1. A. Heatmap of high-confidence YAP1/WWTR1 direct target genes (47) in each expression condition. B. Venn diagrams showing “correction” scheme to obtain DEG whose expression changes were TEAD-dependent following induction of YAP1 or WWTR1. C. RT-qPCR validation of MCPyV *LT* and *ST* transcript levels following induction of wild-type or mutant YAP1 or WWTR1. N = 3; mean + CI calculated from technical replicate SD; normalization to 18S rRNA then GFP; one-way ANOVA with Dunnett post-hoc tests on delta CT values; * $p < 0.05$. D. GOTerm biological process (BP) analysis of two-fold upregulated DEG in corrected WWTR1 and E. YAP1 datasets. F. Volcano plot highlighting the expression of integrin (ITG) and collagen (COL) subunits upon YAP1 or WWTR1 induction compared to GFP.

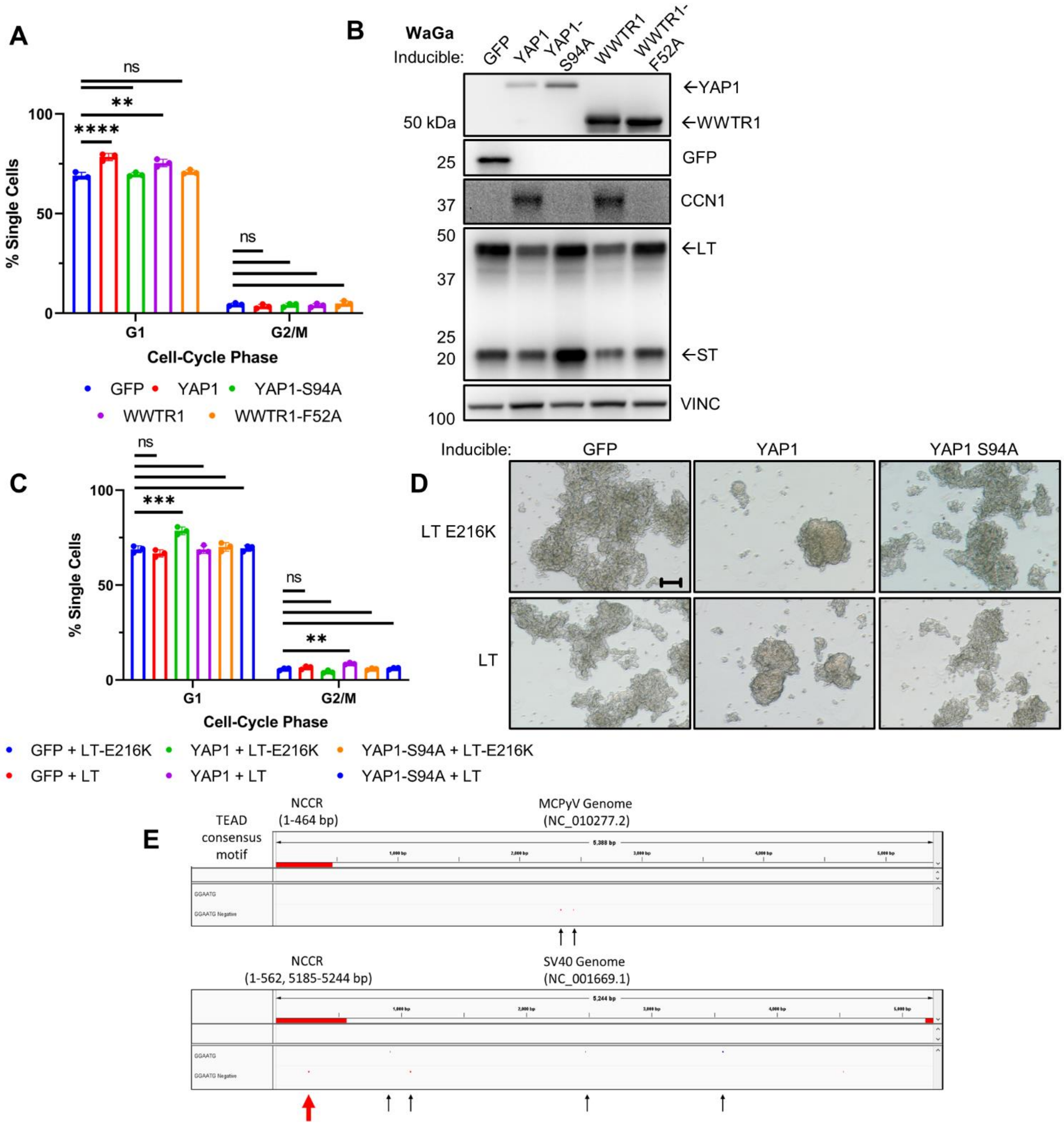

Figure S14. Additional analyses of YAP1/WWTR1 expression in MKL-1 and WaGa cells. A. Quantification of single cells present in the G1 or G2/M phases from cell-cycle analyses of MKL-1 cells induced to express wild-type or mutant YAP1, WWTR1, or GFP constructs for 6 days. N = 3; mean + SD; one-way ANOVA with Dunnett post-hoc tests; ** $p < 0.01$, **** $p < 0.0001$. B. Immunoblot analysis of WaGa cells induced to express wild-type or mutant YAP1 or WWTR1 for 3 days. Representative of 3 biological replicates. C. Quantification of single-cells present in the G1 or G2/M phases from cell-cycle analyses of MKL-1 cells induced to express wild-

type or mutant YAP1 or GFP with constitutive expression of wildtype or RB1-binding defective MCPyV LT for 6 days. N = 3; mean + SD; one-way ANOVA with Dunnett post-hoc tests; ** $p < 0.01$, *** $p < 0.001$. D. Bright-field microscopy of MKL-1 cells with constitutive expression of wild-type or RB1-binding defective MCPyV LT induced to express wild-type or mutant YAP1 for 6 days. 4x magnification; scale bar 100 µm. Representative of 3 experiments. E. Integrative Genomics Viewer (IGV) plot of MCPyV (NC_010277.2) or SV40 (NC_001669.1) genomes highlighted for TEAD-consensus motif (5’-GGAATG-3’). Red bar represents NCCR for either MCPyV or SV40 genomes. Black arrows highlight presence of TEAD consensus motif outside of NCCR, red arrows represent TEAD-binding motif within the NCCR.

Full unedited blots for Fig. 3D

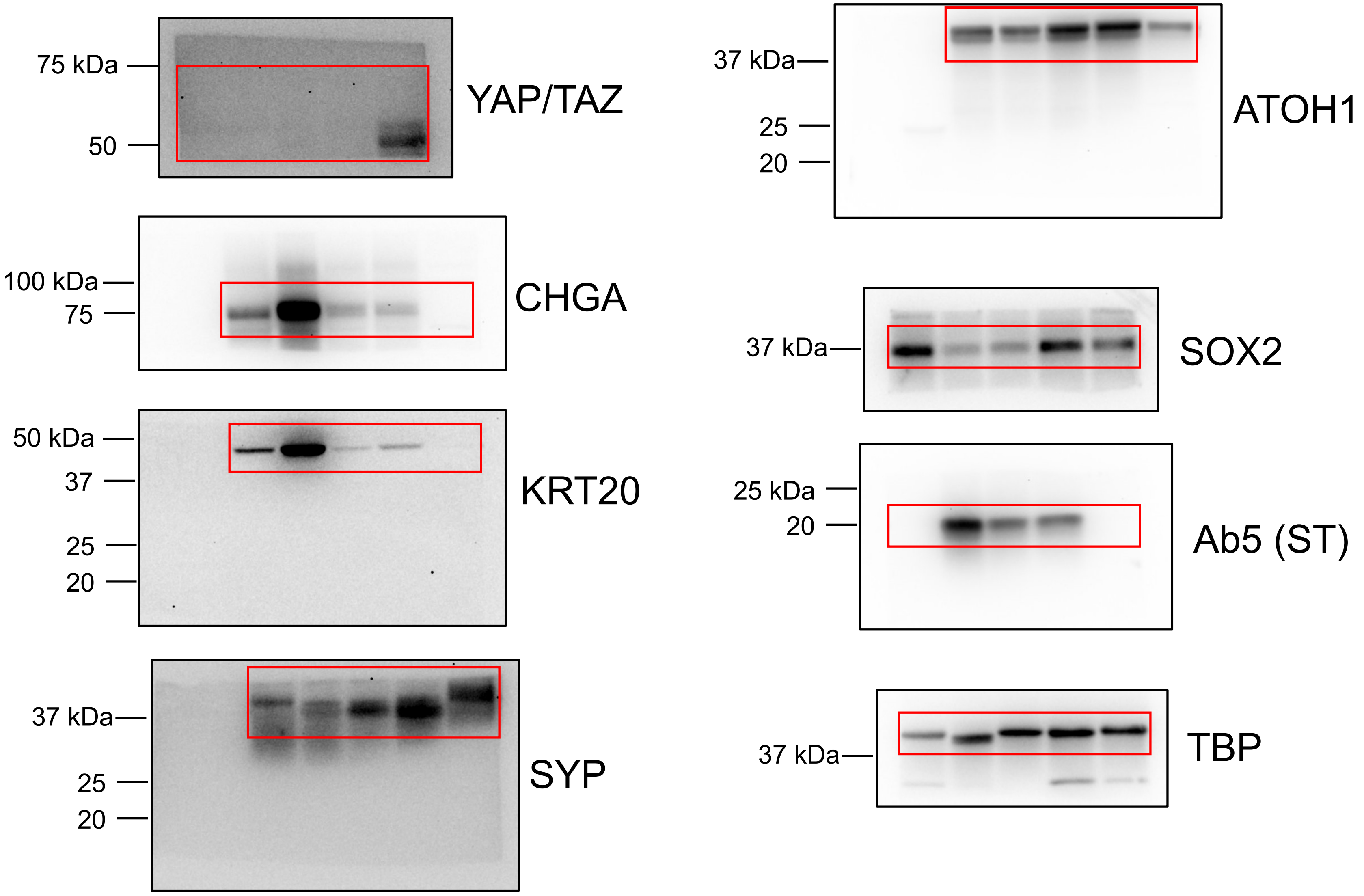

Full unedited blots for Fig. 7B

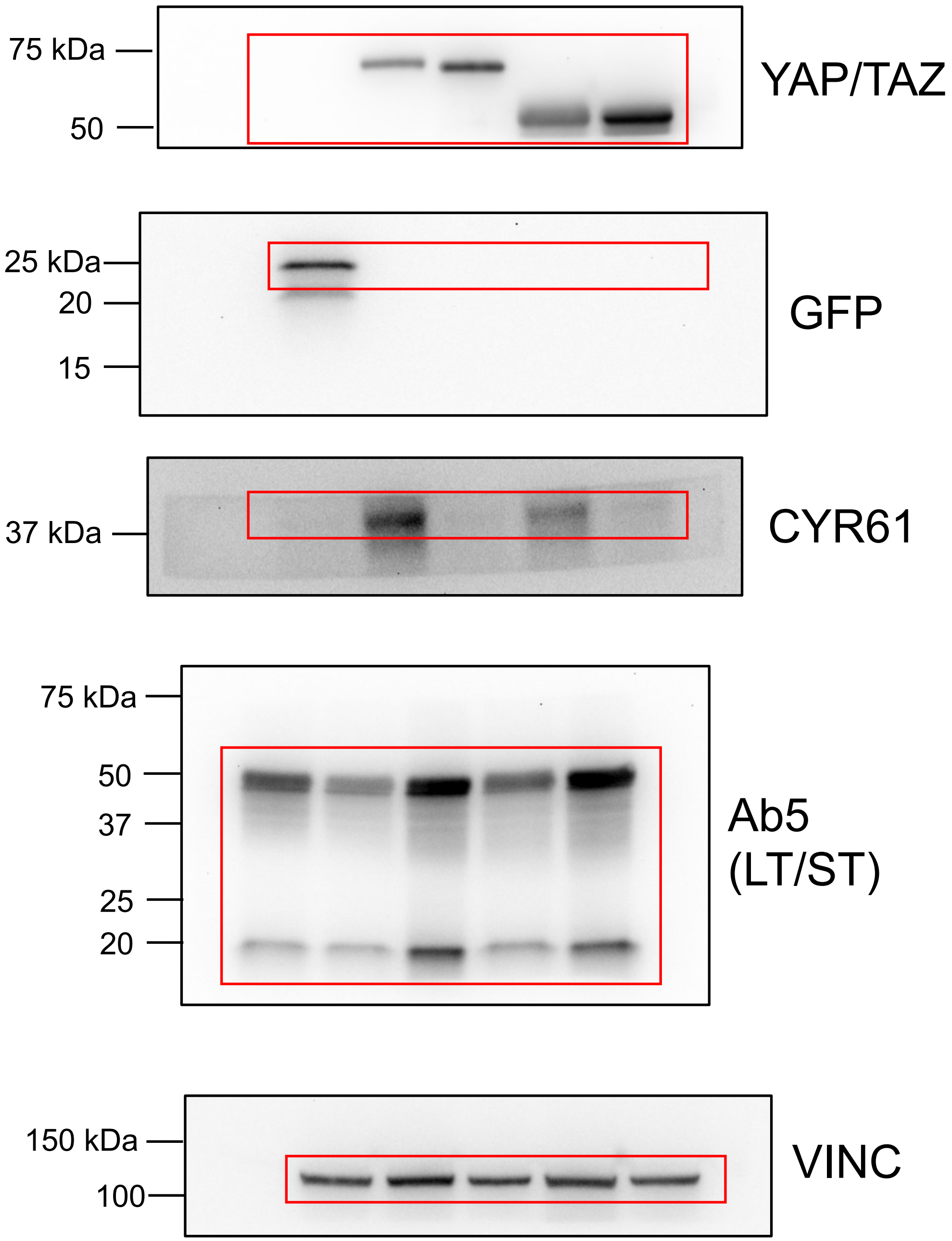

Full unedited blots for Fig. 7E

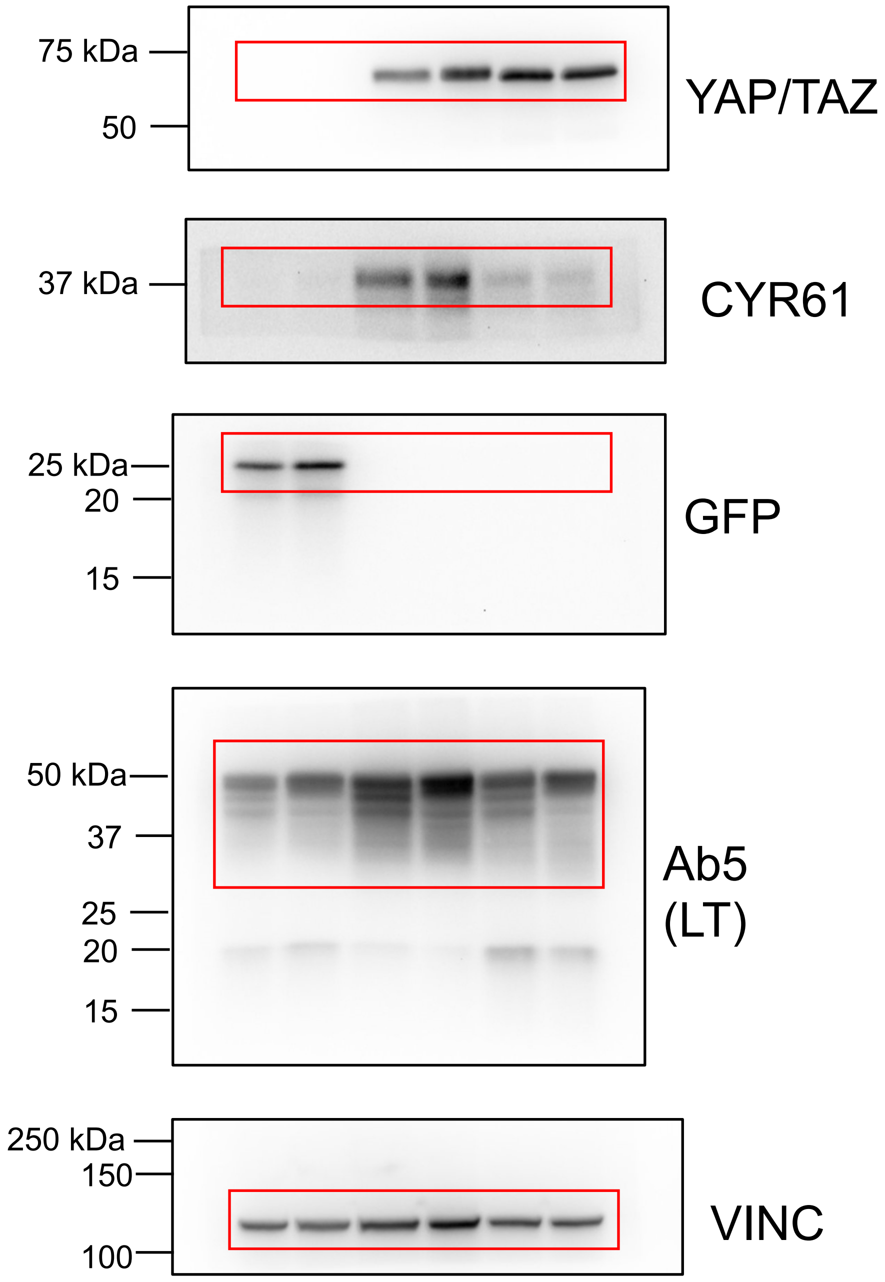

Full unedited blots for Fig. S6B

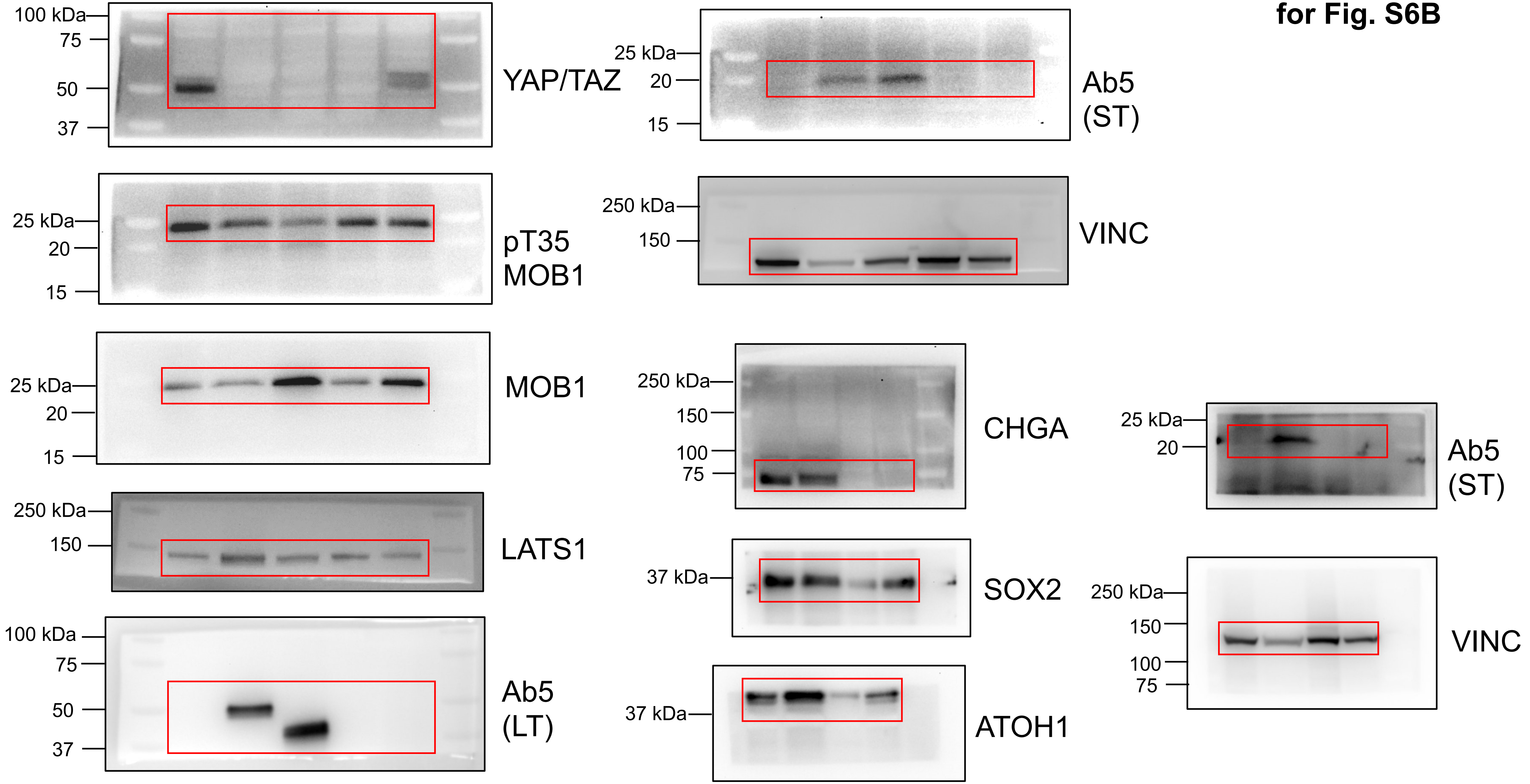

Full unedited blots for Fig. S6D

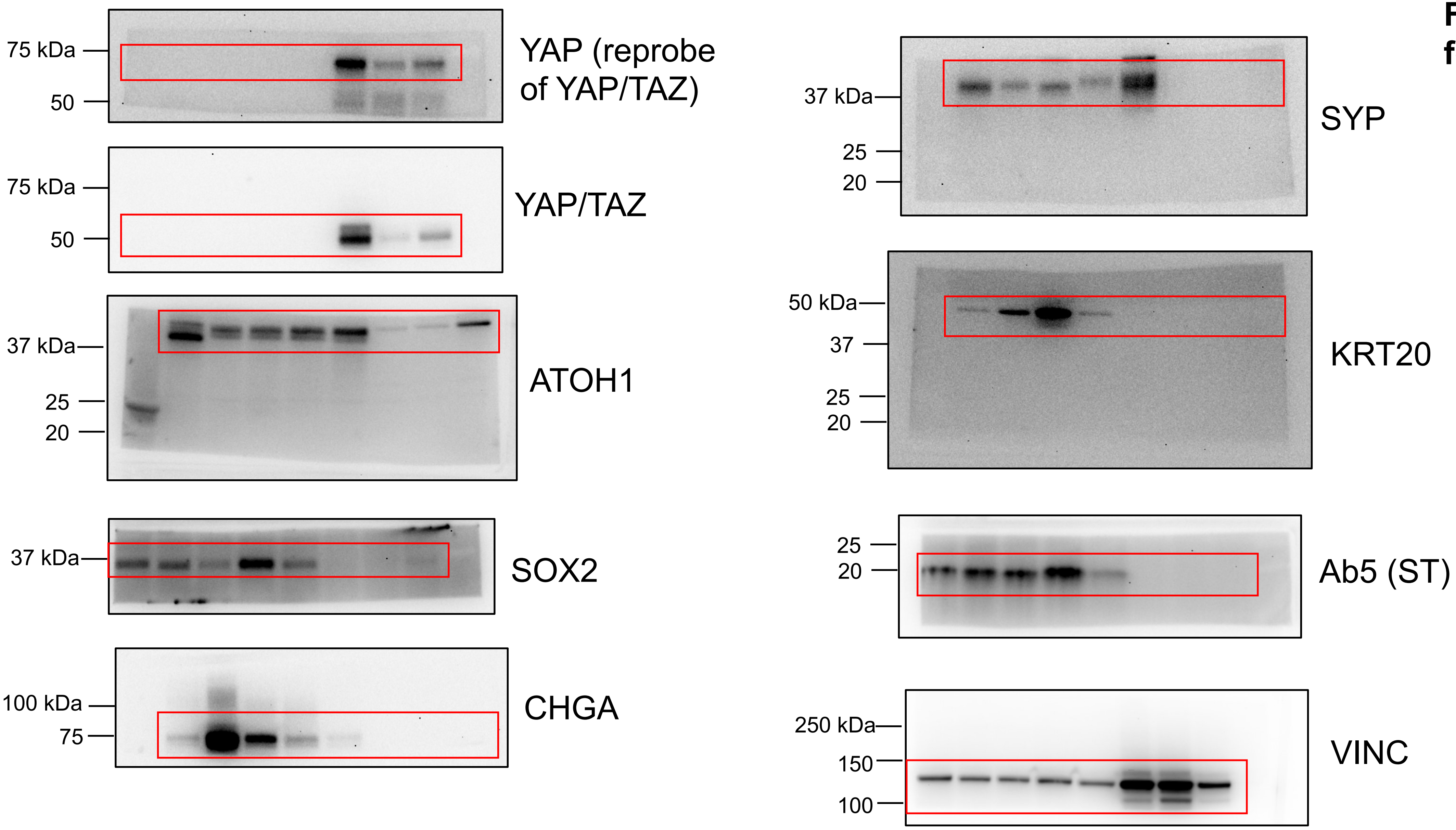

Full unedited blots for Fig. S6E

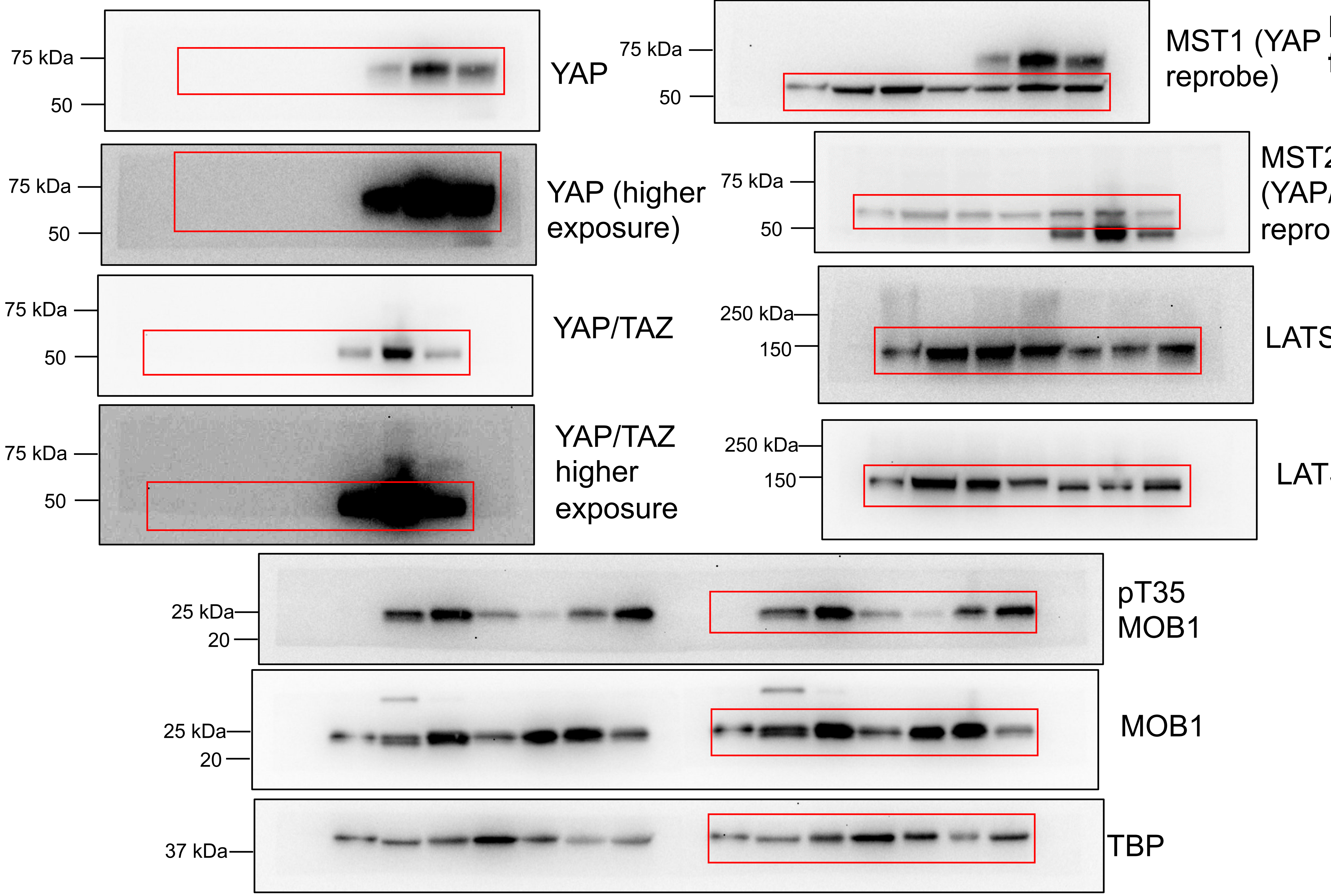

Full unedited blots for Fig. S11A

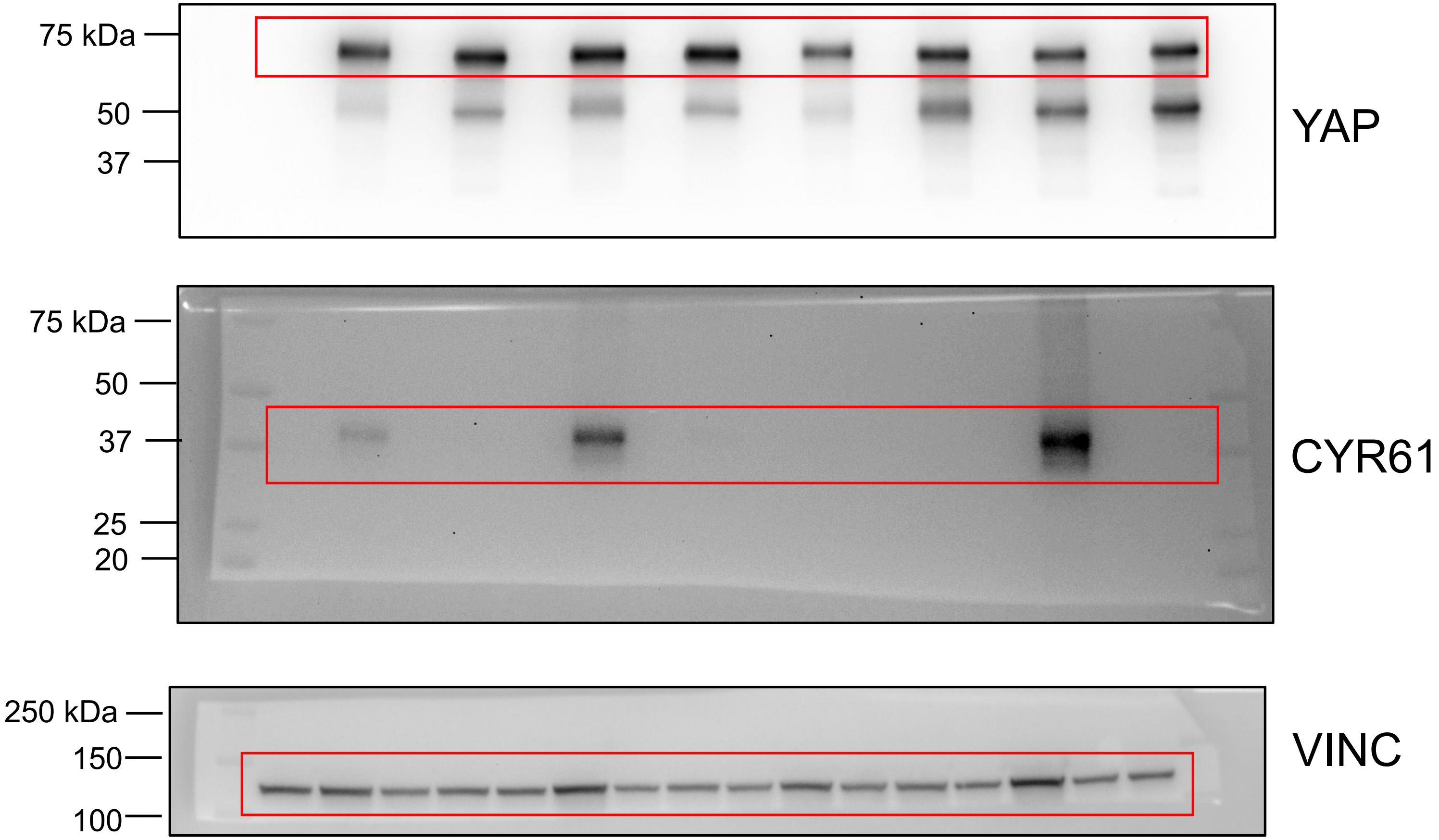

Full unedited blots for Fig. S12A

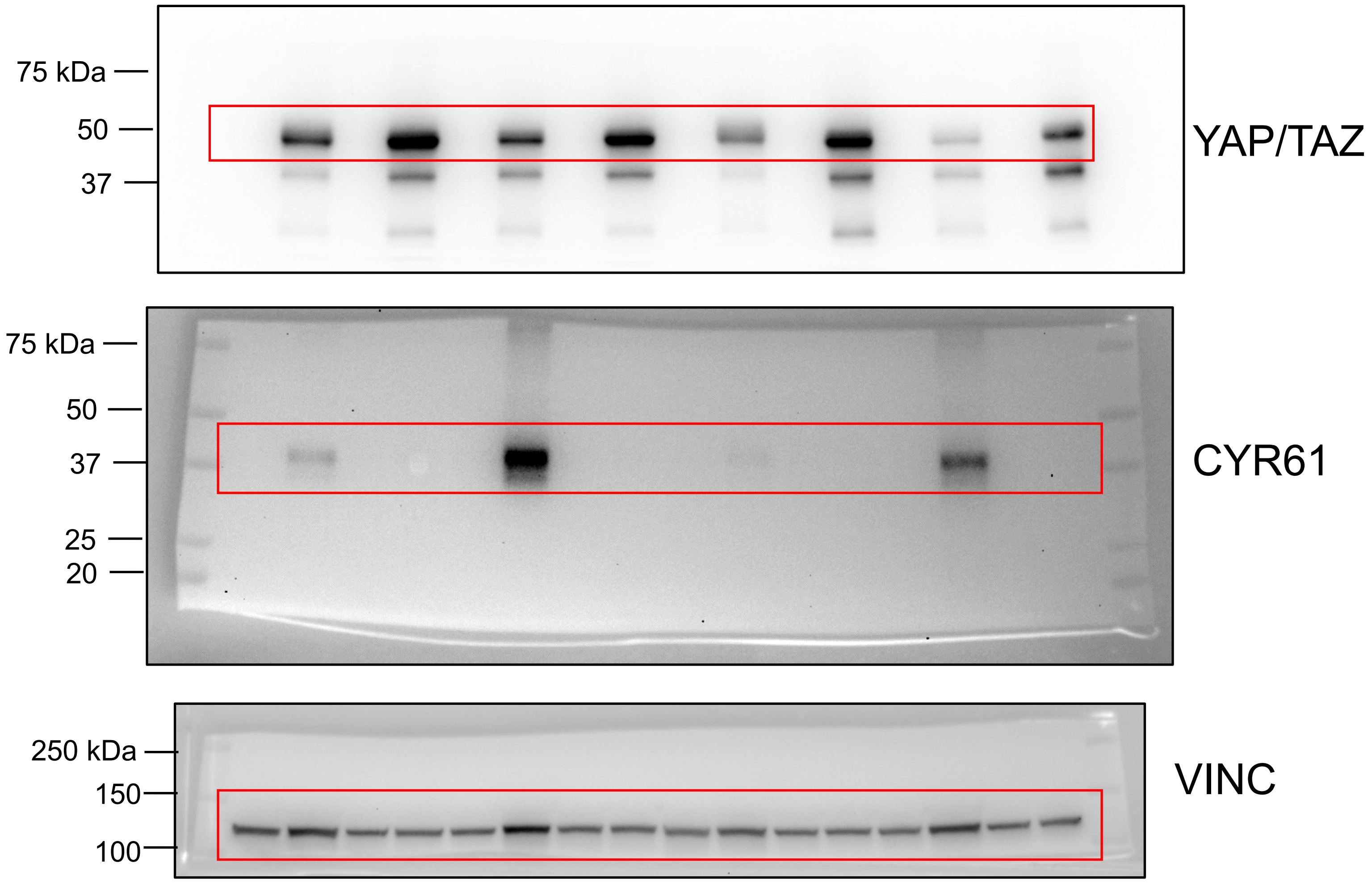

Full unedited blots for Fig. S14B

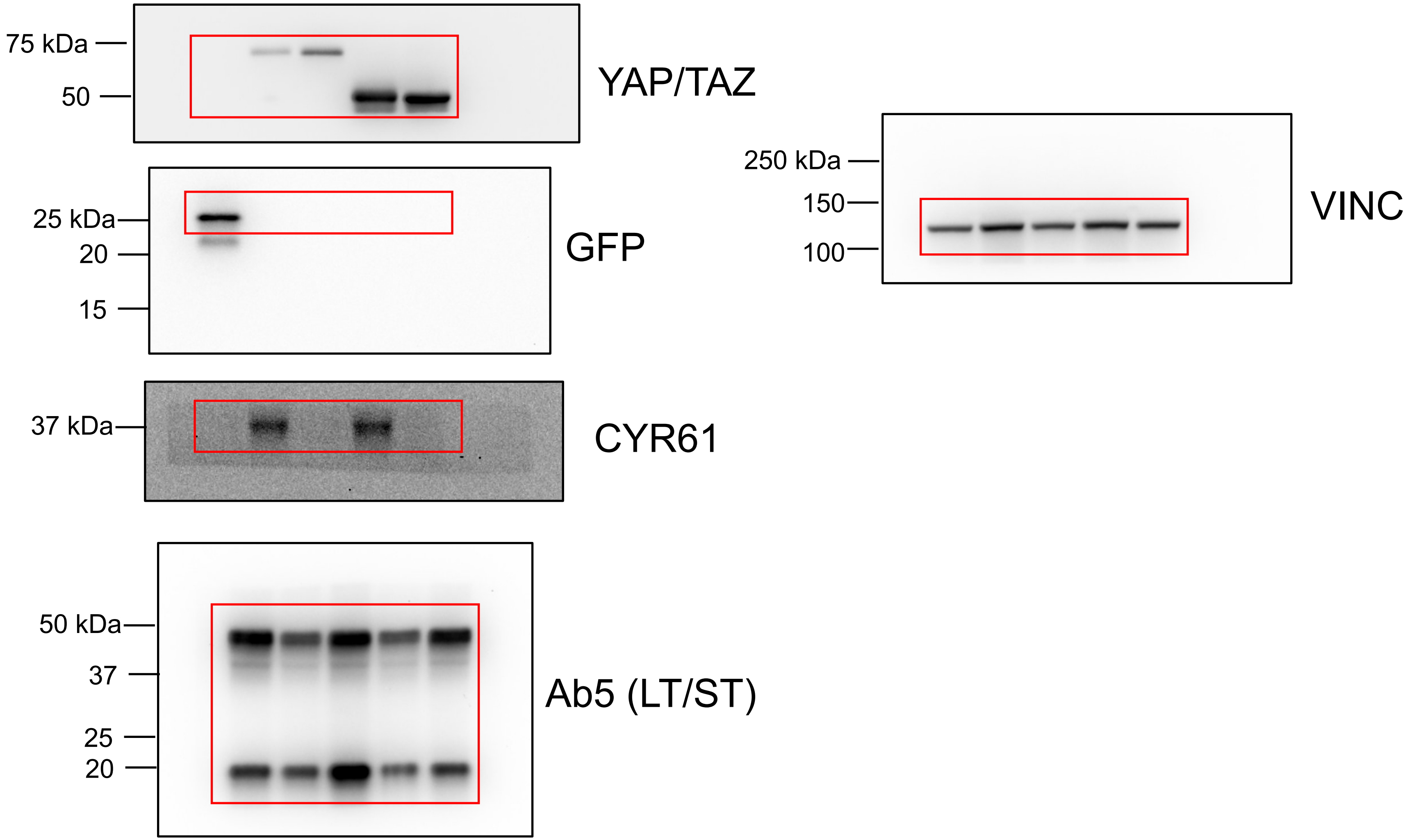

Supplement: Supplemental data [file jci-133-157171-s009.pdf]
