# Supplementary material for: YAP1 and WWTR1 expression inversely correlates with neuroendocrine markers in Merkel cell carcinoma
Source: J Clin Invest. 2023 Mar 1;133(5):e157171. doi: 10.1172/JCI157171 (PMC9974098; doi:10.1172/JCI157171)
Supplement: Trial reporting checklists [file jci-133-157171-s010.pdf]

This report does not contain a clinical trial and thus no reporting checklist is appropriate. It was suggested by the editor (Rexford S. Ahima) to resubmit to the Clinical Research article. Please see the recommendation below.

“In addition to the comments of the reviewers, the Editors ask that you submit your revised manuscript in the Clinical Medicine category (https://secure-
web.cisco.com/1fehFwXscapSPXxwgDRQTinoOLBsMlDVJMR6EcZIU6LRU1BGCn5ml7uPqj6D
k6xroRpqBKPgJFpXNH8oO4dywKZYU_0z63xWiQl3-
aC__VWA4m_YXTtF5aqSKZl9amX2Psa3kddiUwOzthCjyxvLlMUBtE31I6QHERZL7Sxa4q79KG
BrHedLQStCF5RxsM9ZBCmh0qz1FeZyr-r_DP5DwIZtZUBZOFlLVGVLIhxB-
vdQA7i3HF57MlxTowTaL6cljF0-CiC3ZfNemQok1pRmjJIoJkmyX7SbvxGVO0u-
RF9IXE2lQKXtz7WRIdiCrqcLsVF3dNH9BIA0LNFONtwxqpA/https%3A%2F%2Fwww.jci.org%2
Fkiosks%2Fsubmission-revised%23Clinical-medicine”
